# Precision Molecular Sieving Enabled by Tunable Slit-like Nanochannels in Anodic Aluminum Oxide-Supported MXene/GO Composite Membranes

**DOI:** 10.1021/acsami.6c02018

**Published:** 2026-04-14

**Authors:** Mohammad Mozafari, Ali Pournaghshband Isfahani, Saeed Khoshhal Salestan, Manushree Tanwar, Zahra Fakhraai, Masoud Soroush

**Affiliations:** † Department of Chemical and Biological Engineering, 273924 Drexel University, Philadelphia, Pennsylvania 19104, United States; ‡ Department of Mechanical Engineering, 10-241 Donadeo Innovation Center for Engineering, Advanced Water Research Lab (AWRL), University of Alberta, Edmonton, Alberta T6G 1H9, Canada; § Department of Chemistry, 142839 University of Pennsylvania, Philadelphia, Pennsylvania 19104, United States; ∥ Department of Materials Science and Engineering, Drexel University, Philadelphia, Pennsylvania 19104, United States

**Keywords:** Ti_3_C_2_T_*x*_ MXene, graphene oxide, molecular sieving, interlayer spacing, gas separation

## Abstract

Overcoming the permeance-selectivity trade-off in membrane-based separations requires materials with precisely defined nanochannels and robust molecular sieving capabilities. Two-dimensional nanomaterials offer such control; however, random stacking of nanosheets often produces disordered transport pathways that limit separation performance. Here, we report anodic aluminum oxide-supported Ti_3_C_2_T_*x*_ MXene/graphene oxide (GO) composite membranes with tunable interlayer spacing and enhanced sheet alignment for ultrahigh molecular sieving. Large-area GO flakes seal structural defects within the MXene scaffold while inducing well-aligned, slit-like nanochannels. At an optimized MXene/GO weight ratio of 1:1, the composite membranes achieve a H_2_ permeance of 2681.6 GPU (2011.2 GPU under mixed-gas conditions) with a H_2_/CO_2_ selectivity of 536.3 (457.1 under mixed-gas conditions), substantially surpassing the performance of state-of-the-art membranes. Molecular simulations reveal that ordered interlayer galleries and tailored slit pores underpin this exceptional molecular sieving behavior. This scalable composite platform enables high-precision separations for applications such as gas purification, advanced water treatment, and organic solvent nanofiltration.

## Graphical abstract

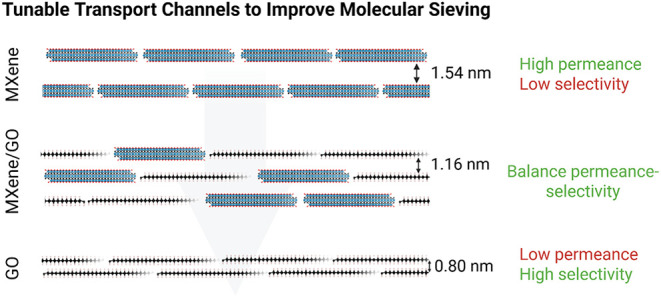

## 1 Introduction

Membrane-based molecular separation has become a cornerstone technology due to its operational simplicity, energy efficiency, and scalability for the separation of gases, ions, and organic molecules.^1–5^ Two-dimensional (2D) materials,such as metal–organic frameworks (MOFs),^6–8^ covalent-organic frameworks (COFs),^9^ zeolites,^10^ transition metal dichalcogenides (TMDs),^11^ transition metal borides (MBenes),^12^ transition metal carbides and/or nitrides (MXenes),^13,14^ graphene, and graphene oxide (GO)^15–17^ , have emerged as versatile building blocks for next-generation membranes. Their tunable interlayer spacing, high aspect ratio, and intrinsic molecular sieving capabilities provide unique opportunities for the selective transport of gases, ions, and organic molecules.^18,19^ In addition, 2D materials exhibit excellent chemical and thermal stability, as well as diverse surface functionalities that can be tailored to a range of separation environments.^20^ Despite these advantages, practical challenges remain: MOFs and zeolites often suffer from structural collapse during exfoliation,^21–23^ COFs require complex and intricate pore engineering,^24^ and TMDs frequently exhibit limited mechanical stability and uncontrolled stacking.^25^

GO, composed of single-atom–thick sheets decorated with hydroxyl, carboxyl, and epoxy groups, exhibits extremely high aspect ratios (>1000) that promote tortuous transport pathways for large molecules while permitting rapid passage of smaller species via size sieving.^26–28^ GO membranes feature two primary transport channels: narrow interlayer galleries and intrinsic structural defects.^29,30^ Consequently, membrane performance depends critically on the nanosheet stacking order and defect density. For example, randomly stacked GO membranes typically exhibit Knudsen-dominated transport, whereas highly interlocked and ordered layers enable molecular sieving with enhanced selectivity.^18^ However, pristine GO membranes alone struggle to simultaneously achieve high permeance and high selectivity. For instance, Ibrahim et al. reported a H_2_ permeance of 1.33 × 10^–7^ mol m^–2^ s^–1^ Pa^–1^ with a H_2_/CO_2_ ideal selectivity of 35.3, attributing the limited separation performance to parallel transport pathways, Knudsen diffusion through defects, and molecular sieving through interlayer channels.^31^ These considerations highlight the central importance of defect sealing and stable, well-defined nanochannels in GO-based membranes.

MXenes (M_*n*+1_X*_n_* T_*x*_, where M is an early transition metal, X is carbon and/or nitrogen, and T_*x*_ represents surface terminations such as −F, =O, and −OH) combine metallic conductivity, hydrophilicity, and mechanical robustness, making them attractive candidates for membrane-based separations.^32–35^ Laminar MXene membranes form interlayer galleries governed by surface terminations and intercalated species, which can yield strong size selectivity when defects are minimized and the gallery structure is controlled.^36^ Ding et al. demonstrated Ti_3_C_2_T_*x*_ membranes with a H_2_ permeability of 2402.3 Barrer and a H_2_/CO_2_ selectivity of 238.4 through molecular sieving,^37^ while Shen et al. reported selectivity improvements from ∼8 to ∼30 through membrane thickness optimization.^38^ These studies underscore that defect mitigation and precise control of interlayer galleries are essential to achieving high performance.

Composite architectures that couple complementary 2D nanosheets provide a practical pathway to improve laminate order and mitigate defects by engineering interfacial packing and transport pathways.^39^ In particular, Ti_3_C_2_T_*x*_/GO composites have been explored extensively in liquid-phase separations (e.g., water nanofiltration, ion/metal-cation sieving, and organic solvent nanofiltration), where performance is frequently linked to composition-dependent control over interlayer spacing, surface chemistry, and electrostatic interactions.^40–42^ Notably, defect-sealing concepts in Ti_3_C_2_T_*x*_/GO laminates have also been emphasized, as incorporating GO can reduce penetration through interedge pathways and strengthen size-selective exclusion.^43^ More recently, cross-linking strategies have enabled angstrom-level control of effective channel spacing and improved structural robustness, reinforcing the importance of stabilizing well-defined galleries in MXene/GO stacks.^44^ Despite these advances, gas-phase transport in Ti_3_C_2_T_*x*_/GO laminates, especially for H_2_/CO_2_ separation, remains comparatively less established, and the respective roles of (i) defect sealing by GO nanosheets, (ii) interlayer gallery constriction, and (iii) MXene–GO interfacial interactions in enabling ultrahigh molecular-sieving selectivity are not yet fully resolved.

Building on these insights, we present Ti_3_C_2_T_*x*_ */*GO composite membranes with engineered interlayer architectures designed to enhance gas-phase molecular sieving performance. By integrating large-area GO flakes into the MXene framework, the composite (i) effectively seals nanosheet defects and interflake voids and (ii) promotes the formation of well-aligned, slit-like nanochannels. At the same time, hydrogen-bonding and electrostatic interactions between MXene terminations and GO functional groups further constrict the interlayer galleries, thereby enhancing size-selective transport. Through systematic structural characterization, gas permeation measurements, and molecular simulations, we demonstrate that an optimized MXene/GO mass ratio of 1:1 yields high H_2_ permeance together with exceptional H_2_/CO_2_ selectivity, placing the membrane among the top reported laminate membranes for hydrogen purification. Although the present study focuses on hydrogen purification, this scalable membrane design paradigm is readily extendable to the precise separation of other gases, ions, and organic contaminants.

## 2 Experimental Section

### 2.1 Materials

GO was obtained from graphene as an aqueous slurry (2 wt % in water). Supplier-provided quality-assurance data are listed in Table S1. Hydrochloric acid (HCl, 12 M) and hydrofluoric acid (HF, 48–51 wt % in water) were purchased from Thermo Scientific and Acros Organics, respectively. Anodic aluminum oxide (AAO) substrates (25 mm in diameter and with a 0.1 μm nominal pore size) were obtained from Whatman. Deionized (DI) water (with a resistivity of ≥ 18 MΩ·cm) was used throughout all experiments. All gas cylinders were supplied by Airgas. Unless otherwise noted, all reagents and materials were used as received without further purification.

### 2.2 Synthesis of Ti_3_C_2_T_*x*_ MXene

For each gram of Ti_3_AlC_2_ MAX phase, 30 mL of etchant was prepared by mixing HF (28.4 M), DI water, and HCl (12 M) in a 1:3:6 (v/v/v) volume ratio.^32^ HCl was added to a 125 mL Nalgene high-density polyethylene (HDPE) bottle prefilled with DI water, followed by the addition of HF. The mixture was placed in an oil bath at 35 °C and stirred at 200 rpm. Ti_3_AlC_2_ MAX phase powder was then added slowly at a rate of 1 g/min. After the addition, the stirring speed was increased to 400 rpm and maintained at 35 °C for 24 h. The resulting acidic slurry was washed by successive centrifugation cycles (5 min at 3234 relative centrifugal force (RCF)) with DI water until the supernatant reached a pH of about 7. To delaminate the neutralized multilayer Ti_3_C_2_T_*x*_ MXene, 50 mL of 0.47 M LiCl solution was added per gram of starting MAX, and the mixture was stirred at 400 rpm and 65 °C for 1 h under an argon atmosphere. The product was then washed via four sequential centrifugation cycles (5, 10, 15, and 20 min at 3234 RCF) with DI water to remove residual salts. Finally, the resulting MXene “clay” was redispersed in DI water, vortex-mixed for 30 min, and centrifuged at 2380 RCF for 30 min to yield a stable suspension of single- to few-layer Ti_3_C_2_T_*x*_ nanosheets for membrane fabrication. Figure 1A illustrates the overall synthesis workflow.

**Figure 1:**
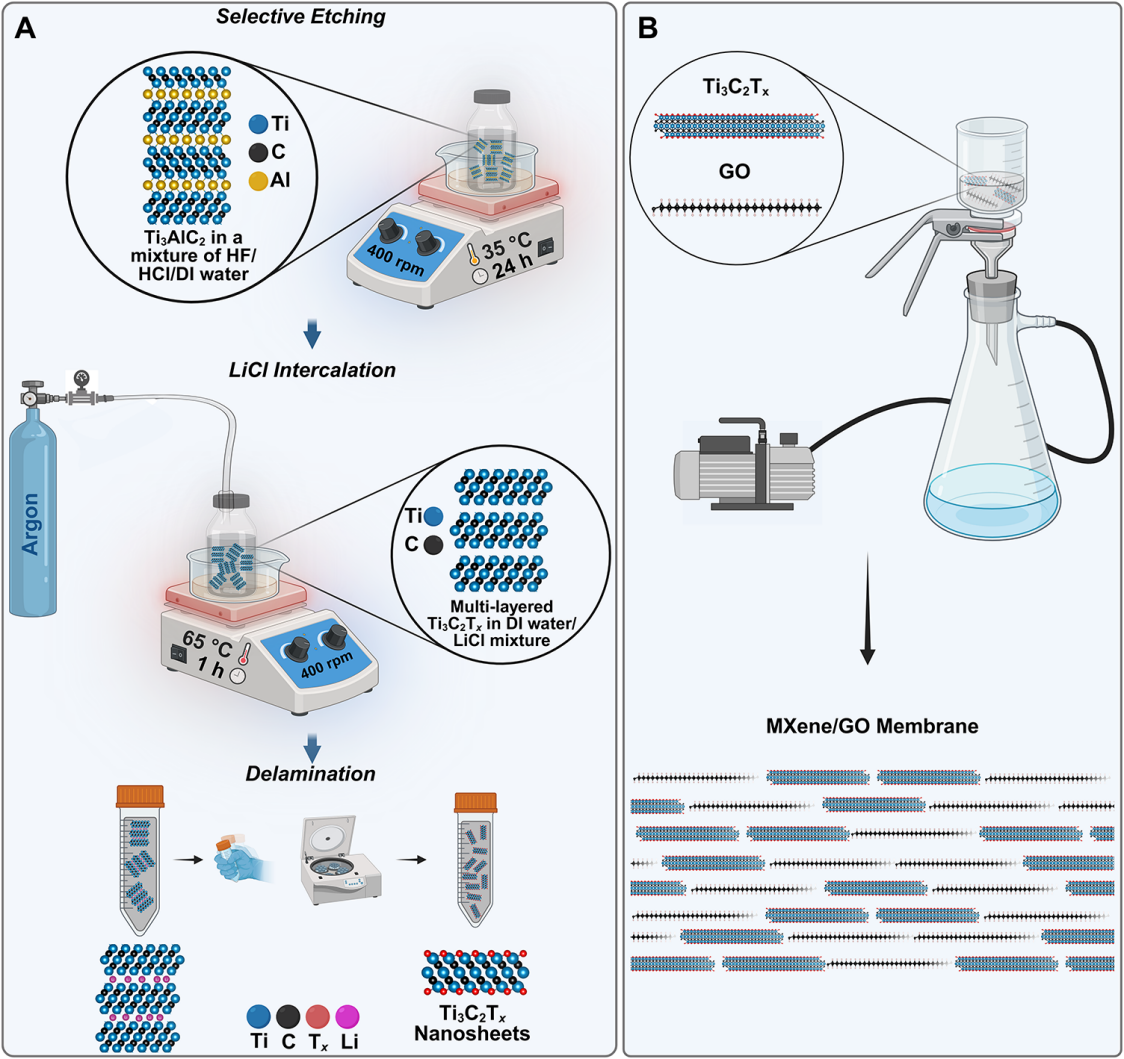
Schematic illustration of (A) the etching and delamination steps for preparing Ti_3_C_2_T_*x*_ MXene nanosheets and (B) the vacuum filtration-based assembly of pristine MXene, pristine GO, and MXene/GO composite membranes on AAO supports.

### 2.3 Preparation of Pristine and MXene/GO Membranes

Prior to the preparation of the MXene/GO casting dispersions, the as-received GO slurry was diluted with DI water, bath sonicated for 10 min, and clarified by low-speed centrifugation to remove large aggregates; only the supernatant was used for subsequent membrane casting, yielding a stable, monolayer-rich dispersion. Membranes were then fabricated via vacuum filtration on AAO substrates. Composite formulations, denoted MXnGOm (n:m = 1:0, 3:1, 1:1, 1:3, 0:1 by mass), were prepared by dispersing a total of 0.5 mg of MXene (MX) and GO in 10 mL of DI water. Suspensions were sonicated for 10 min and stirred at 400 rpm for 1 h to achieve a homogeneous dispersion without visible sedimentation. Each dispersion was then filtered under vacuum (Figure 1B) to deposit the layered film on the AAO substrate. After filtration, the membranes were peeled from the support and dried in a vacuum oven at 60 °C overnight.

### 2.4 Characterizations

The porous structure of pristine and composite membranes was evaluated via H_2_ and CO_2_ adsorption–desorption isotherms using a BELSORPMax analyzer (BEL), yielding pore size distributions, pore volumes, and specific surface areas. Chemical functionalities and surface terminations were probed by Fourier transform infrared spectroscopy (FTIR; Shimadzu IRTracer100, ATR cell, 4000–500 cm^–1^) and X-ray photoelectron spectroscopy (XPS; PHI VersaProbe 5000). Raman spectroscopy (Renishaw in Via Confocal, 785 nm laser) assessed structural ordering in MXene, GO, and composite films, while UV–Vis spectra (Shimadzu UV1700, 10 mm cuvette, 200–1000 nm) monitored optical absorbance.

Morphology and thickness were characterized by atomic force microscopy (AFM; Bruker Icon, tapping mode, 300 kHz) and scanning electron microscopy (SEM; Thermo Fisher Apreo 2S, low vacuum). Elemental distribution was determined by energy-dispersive X-ray spectroscopy (EDS) on the SEM. Crystallographic structure and interlayer spacing were examined by X-ray diffraction (Rigaku RINT, Cu Kα, λ = 1.54 Å, 5–40° 2θ at 40 kV, 40 mA). Transmission electron microscopy (TEM; JEOL 2100F with 50 mm^2^ EDS detector) provided nanoscale imaging of sheet morphology. Finally, nanomechanical properties were measured using an MTS Nanoindenter XP (displacement resolution 0.02 nm; load resolution 50 nN).

### 2.5 Molecular Dynamics Simulation

Classical molecular dynamics (MD) and Monte Carlo (MC) simulations were performed to elucidate transport phenomena in MXene/GO membranes via two complementary approaches: (i) assessing interfacial compatibility between GO and MXene species and (ii) calculating confined diffusion coefficients of CO_2_ and H_2_ within subnanometer channels at GO, MXene, and GO–MXene interfaces. All simulations employed the Universal Force Field (UFF) in Materials Studio, with atomic partial charges assigned via the Charge Equilibration (Qeq) method. Electrostatic interactions were treated using Ewald summation (precision = 10^–5^ kcal/mol). van der Waals forces were handled with an atom-based summation and an 18 Å cutoff. Temperature was maintained at 298 K using a Nosé–Hoover thermostat throughout.

#### 2.5.1 Compatibility between Species in the MXene/GO

To evaluate the interfacial compatibility between Ti_3_C_2_T_*x*_ MXene and GO, and to probe the influence of MXene surface terminations, we performed atomistic simulations on monolayer MXene models bearing O, F, or OH terminations. Unit cells for Ti_3_C_2_T_2_ (T = O, F, OH) were constructed following the crystallographic parameters of Hu et al.^45^ and then expanded in the *x*–*y* plane to generate isolated nanosheets. Each sheet was placed in a simulation box with a 60 Å vacuum along the z direction to eliminate interlayer interactions. Surface atoms of each MXene sheet were subjected to an annealing protocol using a canonical Monte Carlo (Metropolis) algorithm. Five heating–cooling cycles were applied, each comprising 100,000 Monte Carlo steps: the system was heated up to 105 K and then cooled to 10 K. The algorithm monitored how the level of the system’s energy changed as one configuration was replaced with another one. If the energy decreased, indicating a more stable configuration, then the change was accepted. If the energy increased, a decision-making process was considered in which the likelihood of accepting a new configuration was proportional to the energy difference between the states as well as the system’s temperature. This procedure samples low-energy configurations and identifies favorable terminations for MXene–GO interactions. Figure S1 illustrates the relaxed vacuum slabs of O-, F-, and OH-terminated Ti_3_C_2_T_2_, alongside the GO flake model employed in subsequent interfacial simulations.

#### 2.5.2 Calculation of Confined Diffusion Coefficients of Gases within Different Interfaces

MD simulations were carried out to extract diffusion coefficients of CO_2_ and H_2_ within GO, Ti_3_C_2_O_2_ MXene, and MXene/GO nanochannels. All simulations employed periodic boundary conditions and were performed using the canonical (NVT) and isothermal–isobaric (NPT) ensembles with Nosé–Hoover thermostat and barostat controls.

##### 2.5.2.1 GO Membrane Model

Five GO flakes were arranged in a simulation cell (Figure S2A). The system was equilibrated under the NVT at 300 K for 50 ps and then under the NPT at 1 bar and 300 K for 2 ns. The final configuration defined the GO membrane.

##### 2.5.2.2 MXene/GO Composite

Ti_3_C_2_O_2_ MXene monolayers and GO flakes were stacked to form a laminar composite (Figure S2B). This structure was designed to represent a local GO–MXene interfacial environment within the optimized composite membrane rather than the full composition-dependent morphology at different bulk MXene/GO mass ratios. This assembly was equilibrated under NVT (300 K, 50 ps) and NPT (1 bar, 300 K, 100 ps). Only the O-terminated MXene was considered, as it is the predominant surface species.

##### 2.5.2.3 MXene Membrane Model

A single H_2_O molecule was first inserted into the interlayer region of the equilibrated Ti_3_C_2_O_2_ slab (Figure S2C) to adjust the spacing. It was subsequently replaced, one at a time, with CO_2_ or H_2_ to refine the interlayer gap. Four gas molecules were then loaded to yield the final MXene model (Figures S2D and S2E).

##### 2.5.2.4 Diffusion Coefficient Calculation

For each membrane (GO, MXene, and MXene/GO), four CO_2_ or H_2_ molecules were placed in the equilibrated cell. After 100 ps of NVT equilibration at 300 K, a 5 ns NVT run was performed to sample trajectories. The diffusion coefficient DDD was obtained from the slope of the mean-square displacement (MSD) vs time via the Einstein relation^46^

1 $$\mathrm{MSD}\;\left(t\right)=\frac{1}{N}\sum_{i=1}^{N}\left\langle {\left[r_{i}\left(t_{0}+t\right)-r_{i}\left(t_{0}\right)\right]}^{2}\right\rangle =B+6\mathrm{Dt}$$

where *D* is the diffusion coefficient, *t* is the time, *B* is the intercept from linear fitting, *N* is the number of gas molecules, and *r*_*i*_ is the position vector of molecule *i*.

### 2.6 Gas Permeation Experiments

Gas permeation was measured for both pure gases and H_2_/CO_2_ mixtures using a constant-pressure/variable-volume setup (Figure S3) at room temperature. A stainless-steel membrane module (Millipore XX4502500; 25 mm; area = 2 cm^2^) was used. Permeate composition was analyzed online by a Shimadzu GC2014 equipped with a thermal conductivity detector using nitrogen as the carrier gas. For single-gas permeation tests, a feed flow of 25 sccm was maintained on the feed side with a 25 sccm sweep gas flow on the permeate side to transport permeating gases. For H_2_/CO_2_ mixed-gas permeation tests (*x*/(100–*x*) v/v; *x* = 10, 30, 50, 70, 90), the total feed flow was maintained at 50 sccm, with each gas supplied at 25 sccm. Feed flow was controlled via a needle valve and monitored using a Shimadzu flow meter. Both the feed and permeate pressures were maintained at 1 bar. Unless otherwise noted, the gas-separation performance for each membrane type was evaluated using two independently prepared membrane samples and the measurements were repeated to verify reproducibility. Pure-gas permeability *P_i_* was calculated from the following equation

2 $$P_{i}=\frac{l}{\Delta p}\times \frac{273.15}{273.15+T}\times \frac{P_{\mathrm{atm}}}{76}\times \frac{1}{A}\times {\left(\frac{dv}{\mathrm{dt}}\right)}_{i}$$

where *P* is the permeability (1 Barrer = 1 × 10^–10^ cm^3^ (STP) cm cm^–2^ s^–1^ cmHg^–1^), *A* is the effective membrane area (cm^2^), *T* is temperature (°C), $\frac{\mathrm{dv}}{\mathrm{dt}}$ is the rate of change of permeate volume (cmHg) with time (s), *l* is the membrane thickness (cm), Δ*p* is the transmembrane pressure difference (atm), and *P*_atm_ is the atmospheric pressure (atm). Ideal selectivity for a gas pair *i*/*j* is

3 $$\alpha_{i/j}=\frac{P_{i}}{P_{j}}$$

Mixed-gas experiments (H_2_/CO_2_ mixtures) employed a constant-pressure/variable-volume method. Separation factors were determined by

4 $$\alpha_{i/j}=\frac{y_{i}/y_{j}}{x_{i}/x_{j}}$$

where *x*_*i*_ and *y*_*i*_ are the mole fractions of component *i* in the feed and permeate streams, respectively.

## 3 Results and Discussions

### 3.1 Characterization of Ti_3_C_2_T_*x*_ and GO Nanosheets

Minimizing defects in Ti_3_C_2_T_*x*_ MXene is critical for realizing high-performance 2D membranes. Al layers are selectively etched from the Ti_3_AlC_2_ MAX phase using a 1:3:6 (v/v/v) mixture of HF/HCl/DI water (see the Experimental Section). SEM images in Figures 2A,B and S4 reveal the characteristic accordion-like morphology of multilayered Ti_3_C_2_T_*x*_, confirming effective Al removal. Ultrathin GO and Ti_3_C_2_T_*x*_ flakes on Si substrates (Figure S5A and B) appear nearly transparent to the electron beam with straight, well-defined edges and no observable holes or impurities. Both materials bear abundant surface terminations (−OH, =O, −F), imparting negative surface charge and hydrophilicity; stable aqueous suspensions exhibit clear Tyndall scattering at 0.0125 mg mL^–1^ (Figure S6), this effect is attenuated at higher concentrations.

**Figure 2:**
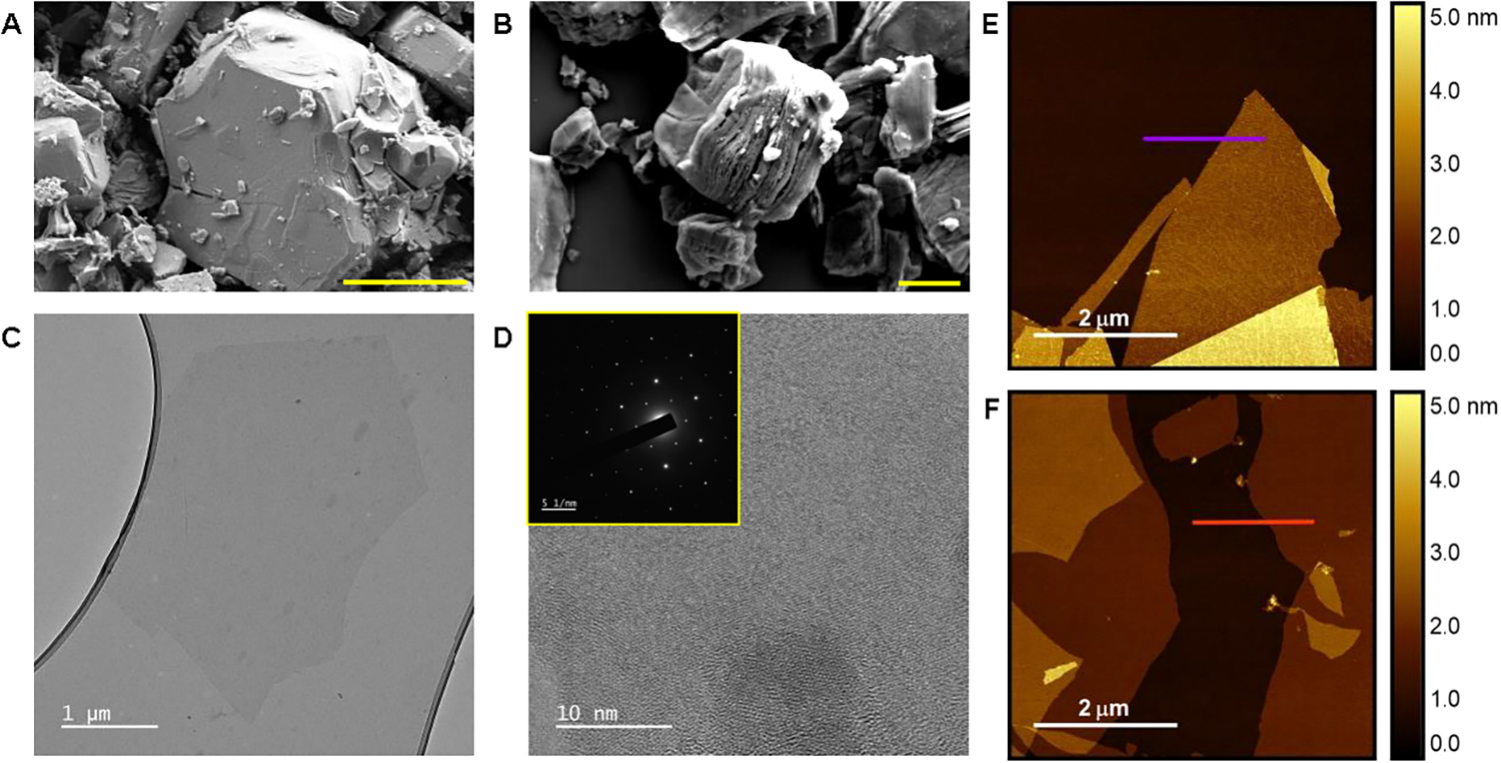
Morphological characterization of Ti_3_C_2_T_*x*_ MXene and GO nanosheets. SEM images of (A) pristine Ti_3_AlC_2_ MAX phase and (B) multilayer Ti_3_C_2_T_*x*_ MXene after etching. TEM images of (C) a single-layer Ti_3_C_2_T_*x*_ nanosheet and (D) high-resolution TEM of Ti_3_C_2_T_*x*_ with the corresponding selected-area electron diffraction (SAED) pattern shown in the inset. AFM topography of (E) Ti_3_C_2_T_*x*_ and (F) GO nanosheets deposited on silicon substrates. The purple and red lines indicate the corresponding height profiles, provided in the Supporting Information (SI).

TEM (Figure 2C) shows atomically thin Ti_3_C_2_T_*x*_ sheets; high-resolution TEM and SAED (Figure 2D) confirm a defect-free, hexagonal lattice. AFM (Figure 2E and F) yields single-layer thicknesses of 1.75 nm for Ti_3_C_2_T_*x*_ and 1.0 nm for GO, values consistent with hydration-expanded monolayers. Corresponding height profiles along the purple and red lines are provided in Figure S7. XRD (Figure S8) shows the disappearance of the Ti_3_AlC_2_ (104) peak at 39° and the emergence of (00l) MXene reflections; the (002) peak shifts from 6.86° to 5.73°, indicating expanded interlayer spacing due to intercalated water and terminations.^32,47^ DLS (Figure S9) gives lateral sizes of 3.1 μm for Ti_3_C_2_T_*x*_ and 4.2 μm for GO, suggesting that larger GO flakes can span and seal MXene defects.^48,49^ Additional GO characterization (UV–Vis, SEM, XPS, and TEM) supplied by the manufacturer is provided in Supporting Note 1 and Figure S10.

### 3.2 Characterization of MXene/GO Membranes

The homogeneous Ti_3_C_2_T_*x*_/GO suspensions were assembled into membranes via vacuum-assisted filtration. During this filtration-driven coassembly, Ti_3_C_2_T_*x*_–Ti_3_C_2_T_*x*_, Ti_3_C_2_T_*x*_–GO, and GO–GO interactions dynamically govern nanosheet restacking. GO nanosheets participate in the coassembly by regulating Ti_3_C_2_T_*x*_ sheet alignment, bridging interflake voids, and suppressing the formation of interfacial defects and slit-like gaps that can arise during laminar stacking. Optical images of the resulting membranes are shown in Figure S11A. Freestanding MX1GO1 membranes can be bent without fracture (Figure S11B and C), indicating good mechanical robustness. SEM images (Figure S12) reveal smooth, defect-free membrane surfaces, while elemental mapping of the MX1GO1 membrane (Figure S13) confirms the presence of surface termination groups. Cross-sectional SEM images of representative membranes (Figures 3A and S14), including pristine MXene, MX1GO1, and pristine GO, reveal continuous and uniformly stacked selective layers on AAO supports with thicknesses in the range of approximately 70–90 nm. Furthermore, the MXene/GO layered structure is clearly visible in the freestanding MX1GO1 (Ti_3_C_2_T_*x*_:GO 1:1 w/w) composite film (Figure 3B), while the corresponding elemental composition is confirmed by elemental mapping in Figure S13. AFM topography (Figure S15 and Table S2) shows smooth, well-ordered surfaces with well-defined interlayer nanogalleries conducive to molecular sieving.

**Figure 3:**
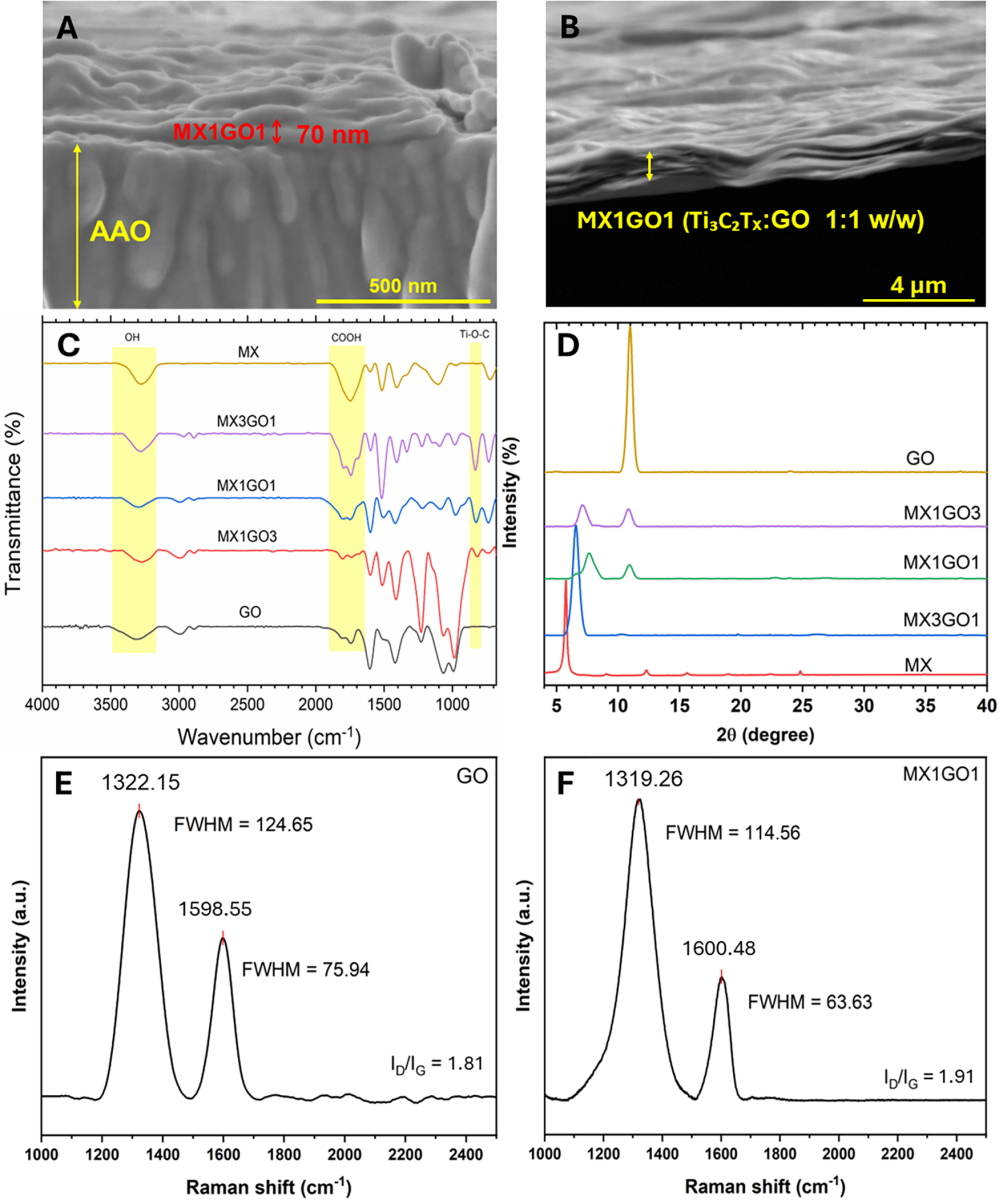
Cross-sectional SEM images of (A) the MX1GO1 membrane on an AAO support and (B) the freestanding MX1GO1 (Ti_3_C_2_T_*x*_:GO 1:1 w/w/) film delaminated from the AAO support. (C) FTIR spectra comparing pristine MXene, GO, and MX1GO1 membranes, highlighting key functional-group signatures. (D) XRD patterns of MXene, GO, and MX1GO1 membranes, showing changes in interlayer spacing upon composite formation. Raman spectra of (E) pristine GO and (F) MX1GO1 membranes, indicating differences in structural ordering and shifts in the D/G bands.

FTIR spectra (Figure 3C) track the functional-group evolution of pristine GO, pristine MXene, and MXene/GO membranes. The characteristic GO peaks at 1078 and 1226 cm^–1^, assigned to C–O–C and C–O stretching vibrations, shift to 1089 and 1231 cm^–1^ in the composite membranes, indicating strong Ti_3_C_2_T_*x*_–GO interfacial interactions through hydrogen bonding between MXene terminations (−F, −OH) and GO’s oxygen-containing groups.^50–52^ Upon addition of MXene, enhanced-COOH/OH stretching and the appearance of a Ti–O–C band at 830 cm^–1^ further corroborate the formation of interfacial bonding.

XRD patterns (Figure 3D) reveal clear structural differences that influence gas transport. The GO membrane shows a peak at 2θ = 10.96°, corresponding to a *d*-spacing of 0.89 nm (Table S3) due to the presence of intercalated water and oxygen-containing functional groups.^53^ Pristine MXene exhibits a well-defined (002) peak at 2θ = 5.73° with a *d*-spacing of 1.54 nm, indicating a highly ordered layered structure suitable for molecular sieving. In MX/GO composite membranes, the (002) peak shifts toward higher diffraction angles, reducing the *d*-spacing to 1.34, 1.16, and 1.24 nm for MX3GO1, MX1GO1, and MX1GO3, respectively. This reduction suggests that incorporating GO alters MXene stacking and promotes a more compact lamellar architecture, which is expected to reduce nonselective interflake voids, thereby enhancing the membrane’s sieving performance.^37^ The *d*-spacing of the membranes was determined using Bragg’s law. After subtraction of the monolayer thickness of the MXene or GO nanosheets, the resulting free interlayer gallery height serves as the molecular-sieving aperture for gas permeation. Collectively, these complementary cross-sectional SEM, XRD, FTIR, and elemental mapping results support the proposed lamellar MXene/GO membrane architecture.

Raman spectra (Figure 3E and 3F) show the characteristic D (1322.15 cm^–1^) and G (1598.55 cm^–1^) bands of GO, indicating structural defects and sp2 hybridized domains.^54^ In the MX1GO1 composite, the D/G features remain prominent, consistent with the incorporation of GO within the laminate. In Ti_3_C_2_T_*x*_–GO composites, the Raman response in this spectral region is typically dominated by GO and has been used to assess GO distribution.^43^ The *I*_D_/*I*_G_ ratio increases from 1.81 (GO) to 1.91 for MX1GO1 (average of six measurement locations, Figure S16), reflecting a modest enhancement in defect/disorder-related scattering in the GO framework after composite formation, consistent with prior reports on MXene/GO membranes.^44^ Overall, the Raman observations suggest changes in the GO local structure within the Ti_3_C_2_T_*x*_/GO laminate and are interpreted in conjunction with the XRD evidence for modified lamellar packing upon composite assembly.^41,55^ The E_g_ and A_1g_ modes of the pristine MXene appear at 290, 362, 593, 198, and 717 cm^–1^ (Figure S17).^56,57^

N_2_ adsorption–desorption isotherms (Figure S18A) exhibit type IV behavior with H_3_ hysteresis loops for all membranes, indicating the presence of narrow slit-like pores.^58^ MXene shows the highest specific surface area (7.56 m^2^/g, Table S4), contributing to the enhanced gas permeance. Upon incorporation of GO, the MX1GO1 membrane displays a significantly reduced average pore diameter (3.94 nm) and reduced pore volume compared to MXene, indicating densification of the laminar structure and suppression of large, nonselective interflake gaps through MXene–GO coassembly. CO_2_ uptake (Figure S18B) is modest but higher in MX1GO1, suggesting preferential CO_2_ adsorption in narrowed galleries.^59^ Taken together, the XRD, Raman, and N_2_ adsorption results collectively indicate structural reorganization and regulated restacking in the MXene/GO membranes.

XPS survey spectra (Figure 4A) confirm the presence of C, Ti, and F in MXene. Introducing GO intensifies the O peak, reflecting its oxygen-rich groups.^32,42^ High-resolution spectra (Figures 4B–D, S19 and S23) show characteristic C–C, C–O, and C=O bonds in GO and Ti–C, Ti–O, and Ti–F bonds in MXene. The TiO_2–*x*_F_2*x*_ content rises from ∼9 at. % (MX) to ∼13–14 at. % (MX1GO1, MX1GO3, MX3GO1), reflecting unavoidable surface oxidation.^60,61^ The preserved peak features confirm the chemical stability and successful integration of the MXene and GO nanosheets.

**Figure 4:**
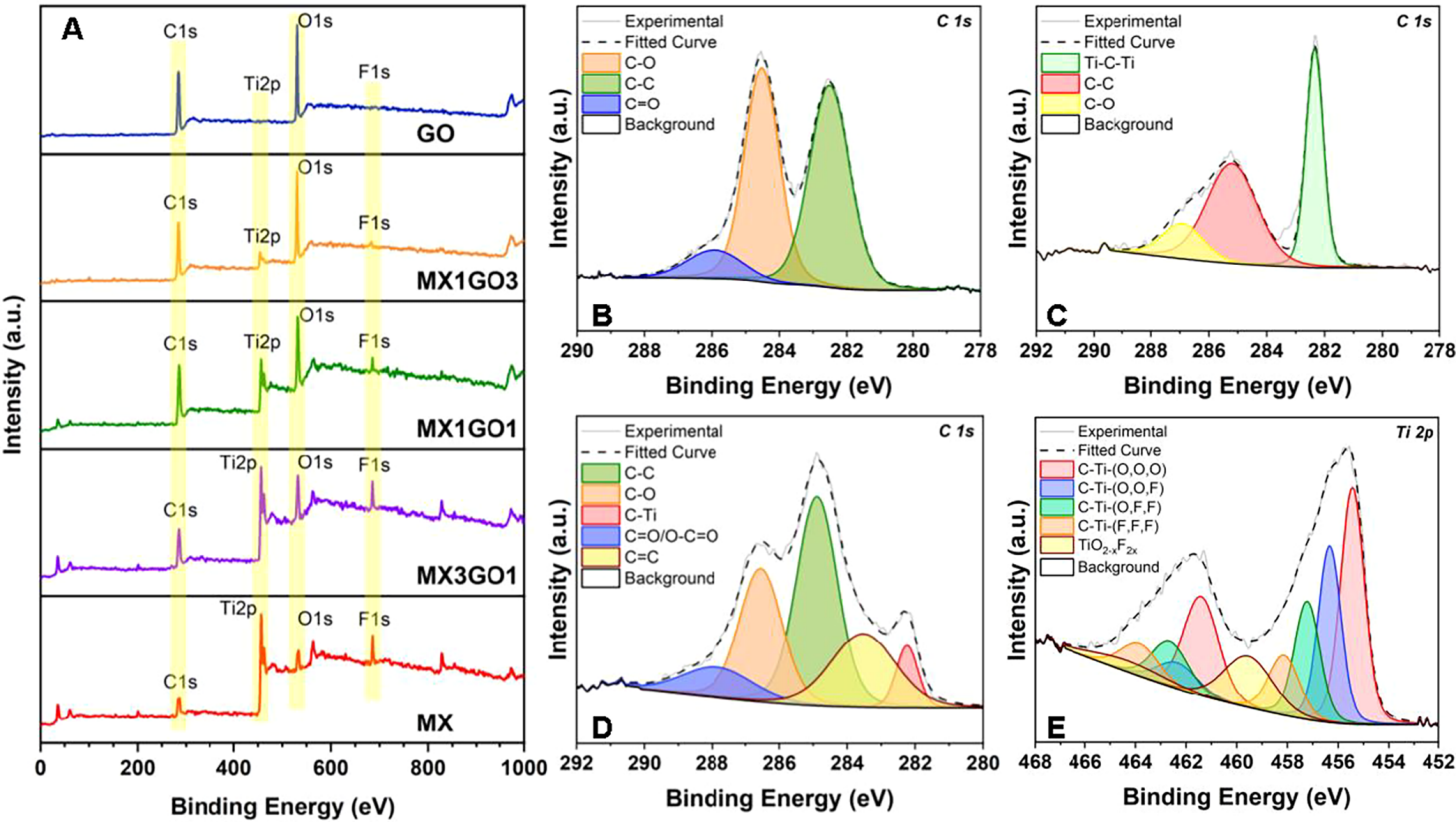
XPS characterization of pristine and MXene/GO composite membranes. (A) XPS survey of MX, MX3GO1, MX1GO1, MX1GO3, and GO membranes. High-resolution C 1s spectra of (B) pristine GO, (C) pristine MXene, and (D) the MX1GO1 composite, showing changes in carbon bonding environments. (E) High-resolution Ti 2p spectrum of the MX1GO1 composite, confirming the presence of Ti–C and Ti–O bonding environments.

### 3.3 Gas-Transport Behavior through Membranes

Gas separation performance of the pristine and MXene/GO composite membranes was evaluated at room temperature. The pure-gas-separation performance of the pristine and MXene/GO composite membranes (Figure 5A,B and Table S5) demonstrates excellent molecular sieving properties. Although CO_2_ has a smaller kinetic diameter than CH_4_, its lower permeance suggests a trapping effect caused by oxygen-containing groups on the MXene and GO. The MX1GO1 membrane exhibits an outstanding H_2_/C_2_H_4_ selectivity of 865.1, underscoring its subnanochannel precision. As shown in Figure 5B, the pristine MX membrane yields high H_2_ permeance (7850.4 GPU) but limited selectivity, while the pristine GO membrane provides lower permeance with higher selectivity. Incorporating GO into the MXene decreases H_2_ permeance but increases the ideal H_2_/CO_2_ selectivity to 536.3 for MX1GO1 (Figure 5B). This performance enhancement is attributed to the formation of an ordered 2D laminar structure, where in-plane slit-like pores govern selective H_2_ transport. These findings confirm that tuning interlayer spacing and pore architecture leads to membranes with an optimized permeance–selectivity balance.^15,20,62–64^

**Figure 5:**
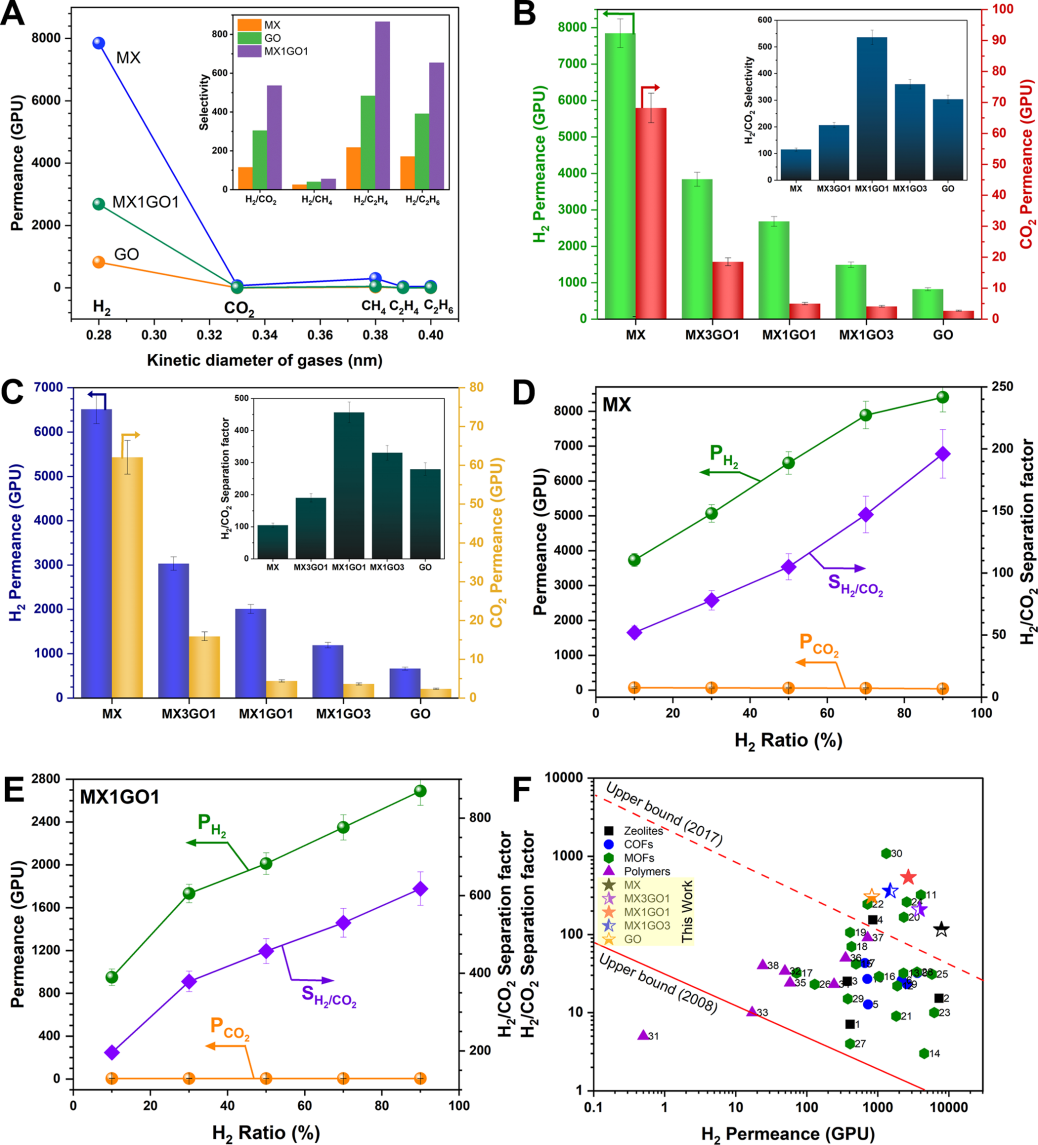
Gas-separation performance of H_2_-selective pristine MX, pristine GO, and MX/GO membranes. (A) Pure-gas permeances of H_2_, CO_2_, CH_4_, C_2_H_4_, and C_2_H_6_; the inset shows H_2_/gas ideal selectivities. (B) Comparison of pure-gas permeation performance; the inset shows H_2_/CO_2_ selectivity and (C) mixed-gas (H_2_/CO_2_ 50/50%) permeation through each membrane, the inset shows H_2_/CO_2_ separation factor. (D–E) Mixed-gas H_2_/CO_2_ separation performance of (D) pristine MXene and (E) MX1GO1 membranes at varying feed ratios. (F) Robeson-type plot comparing H_2_/CO_2_ separation metrics of pristine MXene, pristine GO, and MXene/GO composites against state-of-the-art membranes (see details in Supporting Information, Table S6).

Mixed-gas (50:50 H_2_/CO_2_) permeation results (Figure 5C) confirm the superior H_2_/CO_2_ separation performance of the MX1GO1 membrane, achieving a H_2_ permeance of 2011.2 GPU and a separation factor of 457.1, which is 4.3 times higher than that of the MX membrane and 1.7 times higher than that of the GO membrane. This highlights the membrane’s precise molecular-sieving capability. As shown in Figure 5D and 5E, increasing the H_2_ concentration in the feed enhances H_2_ permeance due to a stronger driving force, while the CO_2_ permeance decreases, resulting in a selectivity approaching 600 at 90% H_2_. Figure 5F shows that the MX1GO1 membrane offers both high H_2_ permeance (∼2680 GPU) and excellent H_2_/CO_2_ selectivity (∼536), surpassing the 2008 Robeson upper bound. A quantitative comparison with representative H_2_/CO_2_ membranes is summarized in Table S6, and a qualitative comparison highlighting structural-control strategies and transport behavior relative to prior GO/MXene composite membranes is provided in Table S7. These results are attributed to optimized in-plane slit-like pores, reduced *d*-spacing, and the presence of subnanometer channels in the MXene/GO structure. Systematic temperature-dependent permeation measurements are beyond the scope of this study and warrant further investigation in future work.

Although AAO substrates are not widely used in industrial gas separation, they provide a mechanically rigid and chemically inert model support platform with a well-defined and uniform pore geometry. The use of AAO in this study minimizes potential complications associated with support deformation, swelling, or plasticization, enabling a systematic and reproducible evaluation of the Ti_3_C_2_T_*x*_/GO selective layer. From a scalability perspective, the membrane configuration demonstrated here is not intrinsically limited to the AAO and could be adapted to scalable porous polymeric supports or hollow-fiber platforms, which represent an important direction for future investigation.

Atomistic simulations (Figure 6) demonstrate strong hydrogen bonding (<3 Å) between GO functionalities and MXene terminations, supporting favorable interfacial interactions between the two nanosheets. These interactions are expected to enhance structural integrity and eliminate the likelihood of nonselective microvoids, enabling effective molecular sieving and improved H_2_/CO_2_ selectivity.^65^ Interactions between GO and MXene nanosheets modulate the interlayer spacing, affecting nanochannel size and gas transport. Calculated confined diffusion coefficients (Table S8) show that H_2_ diffuses significantly faster than CO_2_, especially at the GO–MXene interface, due to H_2_’s smaller size and lower polarity. This interface yields a high diffusion selectivity of 209.6, highlighting its role in enhancing H_2_/CO_2_ separation. Notably, these confined diffusion coefficients do not directly represent the overall membrane permeance. Instead, they provide local mechanistic insight into transport preferences within the GO–MXene interfacial environment. Our intention was not to reproduce the experimental membrane structure in all its complexity but rather to use simplified molecular models to isolate and understand the local interfacial interactions that may contribute to changes in membrane structure and, consequently, to the observed separation behavior. Accordingly, the simulations were designed as representative models of the local GO–MXene interfacial environment and should not be interpreted as direct reproductions of the full membrane morphology at different bulk MXene/GO mass ratios. The effect of composition on channel size and transport behavior was evaluated experimentally by XRD and gas permeation measurements, while the simulations were used in a complementary manner to probe local interfacial interactions and confined diffusion behavior. The experimentally observed selectivity is expected to arise from the coupled effects of size exclusion, adsorption/affinity, pathway tortuosity, and the membrane’s hierarchical transport configuration.

**Figure 6:**
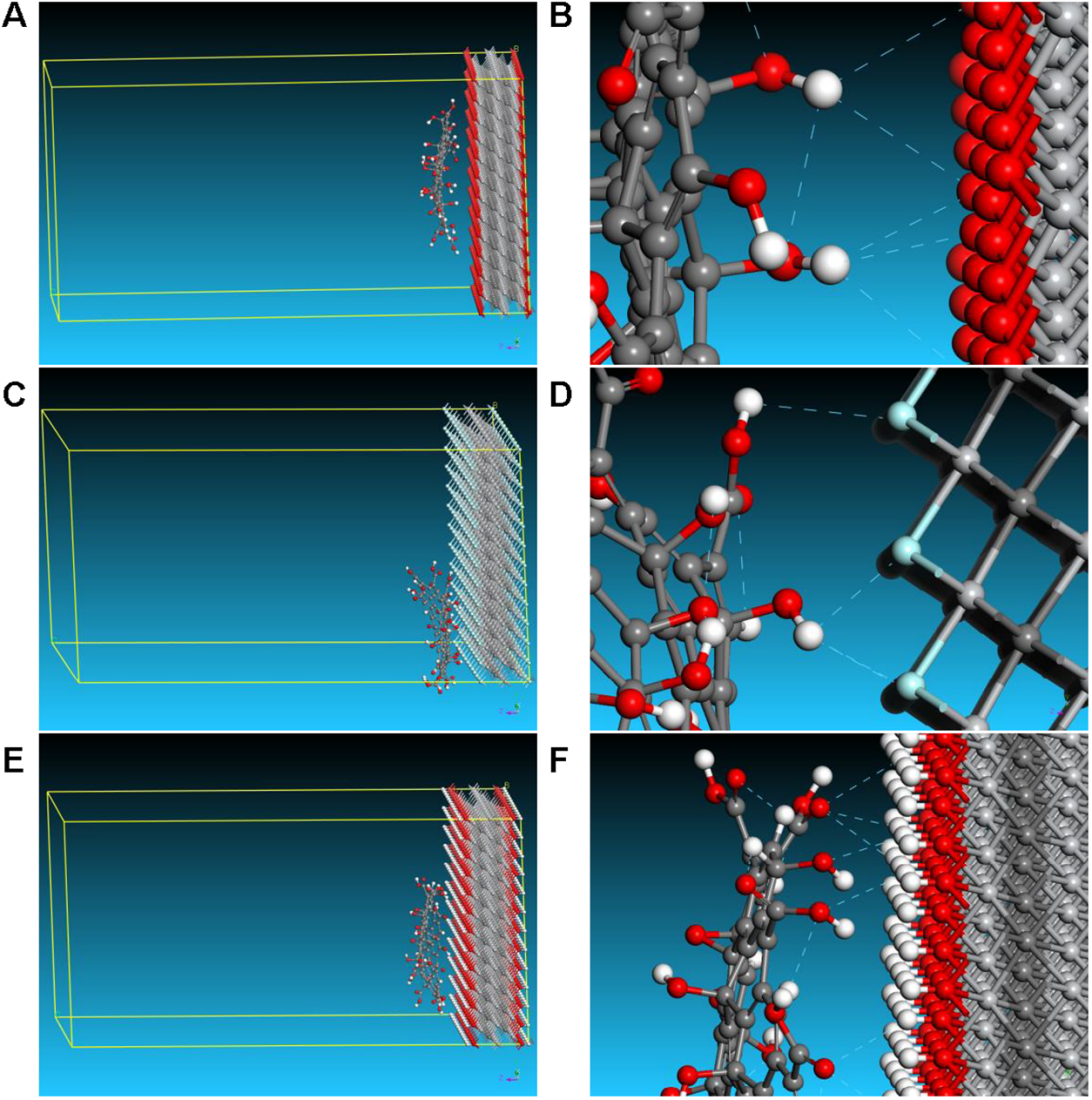
Molecular models illustrating GO–MXene interfacial configurations and hydrogen-bond networks. (A, B) Optimized GO/Ti_3_C_2_O_2_ interface showing GO flakes hydrogen-bonded to =O terminations. (C, D) GO/Ti_3_C_2_F_2_ interface illustrating interactions with – F terminations. (E, F) Ti_3_C_2_(OH)_2_ interface depicting extensive H-bonding to −OH groups. Color legend: Ti (light gray), C (dark gray), O (red), H (white), N (dark blue), F (light blue).

Figure S24 illustrates gas-transport pathways through in-plane slit pores, out-of-plane interlayer galleries, and GO–MXene interfaces within the laminar membrane architecture. In these laminates, the high H_2_/CO_2_ selectivity is primarily governed by molecular sieving enabled by a more ordered stacking structure and suppression of nonselective leakage pathways, rather than by diffusivity differences alone. The effective transport length scales are therefore controlled by the laterally confined galleries and the chemically heterogeneous GO–MXene interfaces formed within the ordered 2D stack, in which the smallest gas (H_2_) experiences the lowest steric resistance. The higher CH_4_ permeance relative to CO_2_ (despite CH_4_’s larger kinetic diameter) is consistent with a strong role of gas–surface interactions: CO_2_ can exhibit stronger affinity (e.g., quadrupolar interactions) with polarized/functionalized MXene/GO surfaces, which can reduce its mobility and increase its residence time within the membrane. Overall, the separation performance can be rationalized by the combined effects of size exclusion through subnanometer transport pathways, defect suppression, and selective adsorption, with interfacial diffusion trends providing supportive insight into transport preferences within confined regions.

## 4 Conclusions

We engineered lamellar Ti_3_C_2_T_*x*_/GO composite membranes in which large GO flakes seal MXene defects and induce well-aligned, slit-like nanochannels. At a 1:1 MXene/GO weight ratio, the membranes achieve a H_2_ permeance of 2681.6 GPU and an ideal H_2_/CO_2_ selectivity of 536.3, improvements of ≈365% over pristine MXene and ≈76% over pristine GO, while mixed-gas measurements yield a separation factor of 457.1 under 50:50 feed conditions. Although adding GO modestly reduces pure-gas H_2_ permeance relative to pristine MXene, the composite still outperforms pure GO by more than 3-fold, striking an optimal balance between permeance and selectivity across a wide range of feed compositions. Molecular simulations reveal that strong hydrogen-bonding and electrostatic interactions at MXene–GO interfaces eliminate nonselective voids and fine-tune interlayer spacing, underpinning the observed molecular sieving. By harmonizing in-plane slit pores, out-of-plane interlayer galleries, and interfacial diffusion pathways, this facile composite strategy establishes a new benchmark for 2D membrane performance. The reported gas-separation performance was reproducible across independently prepared membrane samples, and repeated permeation measurements confirmed consistent separation behavior. Beyond H_2_ purification, where scalable deployment could benefit ammonia synthesis, methanol steam reforming, and carbon capture processes, this tunable nanochannel approach offers a versatile platform for the high-precision separation of ions, other gases, and organic contaminants across gas and liquid media.

## Supplementary Material



## Notes

Schematic of the gas-separation setup; and additional characterization results, SEM, EDS, AFM, XRD, XPS, Raman, DLS, and adsorption isotherms, presented as figures and tables (PDF)
