# Supplementary material for: Precision Molecular Sieving Enabled by Tunable Slit-like Nanochannels in Anodic Aluminum Oxide-Supported MXene/GO Composite Membranes
Source: ACS Appl Mater Interfaces. 2026 Apr 14;18(16):23227–38. doi: 10.1021/acsami.6c02018 (PMC13133784; doi:10.1021/acsami.6c02018)
Supplement: Supplementary file 1 [file am6c02018_si_001.pdf]

Supporting Information (SI)

Precision Molecular Sieving Enabled by Tunable Slit-Like Nanochannels in Anodic Aluminum Oxide–Supported MXene/GO Composite Membranes

Mohammad Mozafari[1,a], Ali Pournaghshband Isfahani[1,a], Saeed Khoshhal Salestan[2], Manushree Tanwar[3], Zahra Fakhraai[3], Masoud Soroush[1,4,*]

[1] Department of Chemical and Biological Engineering, Drexel University, Philadelphia, Pennsylvania 19104, USA

[2] Department of Mechanical Engineering, 10-241 Donadeo Innovation Center for Engineering, Advanced Water Research Lab (AWRL), University of Alberta, Edmonton, Alberta, T6G 1H9, Canada

[3] Department of Chemistry, University of Pennsylvania, Philadelphia, Pennsylvania, 19104, USA

[4] Department of Materials Science and Engineering, Drexel University, Philadelphia, Pennsylvania 19104, USA

Submitted for publication in *ACS Applied Materials & Interfaces*

THIRD REVISED VERSION

April 8, 2026

Keywords: $Ti_3C_2T_x$ MXene; graphene oxide; molecular sieving; interlayer spacing; gas separation

[a] Mohammad Mozafari and Ali Pournaghshband Isfahani contributed equally to this work.

* Corresponding author: soroushm@drexel.edu

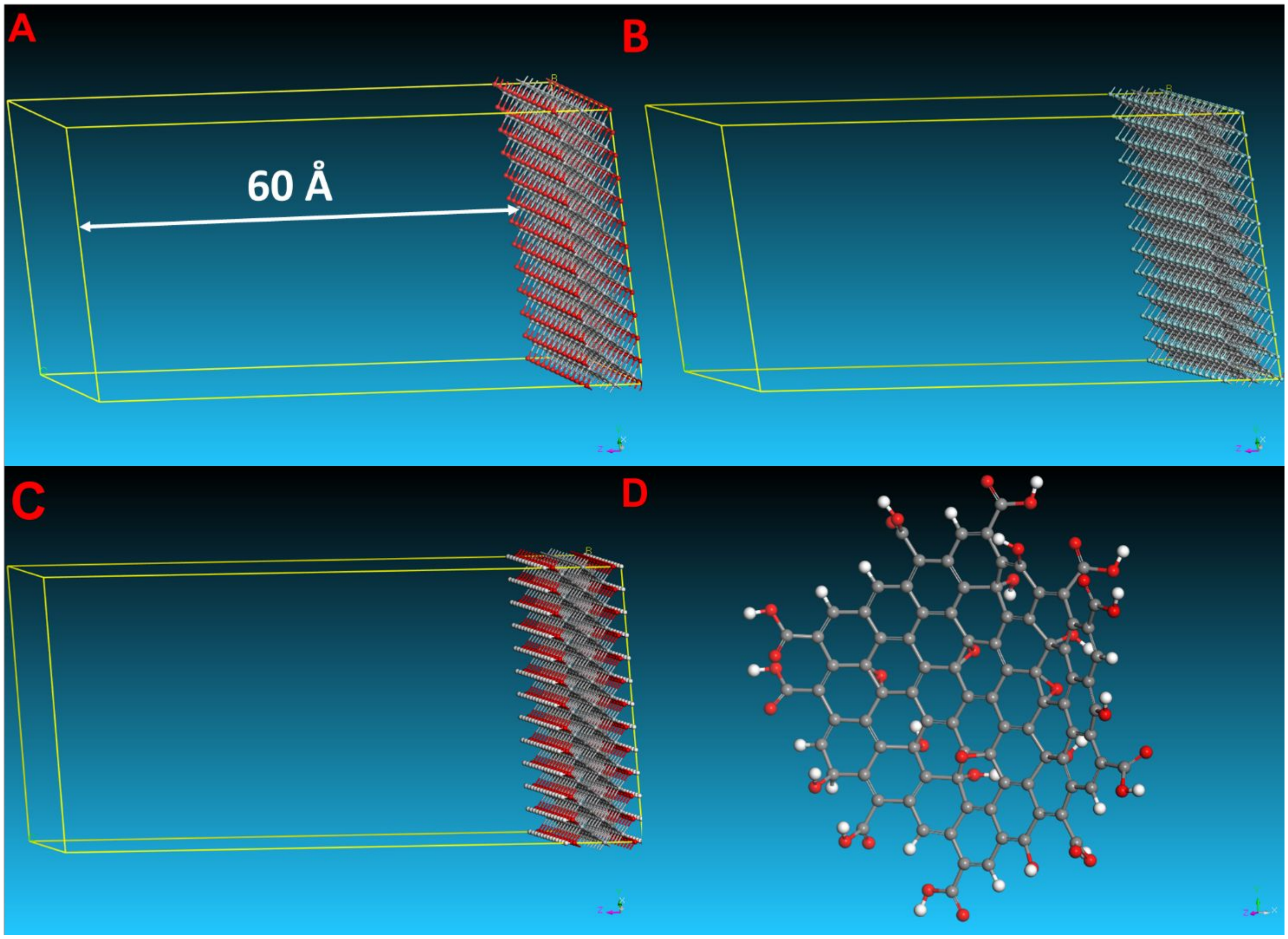

Figure S1. Vacuum-slab-models of MXene with different terminal groups and a GO flake: (A) $Ti_3C_2O_2$, (B) $Ti_3C_2F_2$, (C) $Ti_3C_2(OH)_2$, and (D) GO. Color legend: Ti (light gray), C (dark gray), O (red), H (white), N (dark blue), F (light blue).

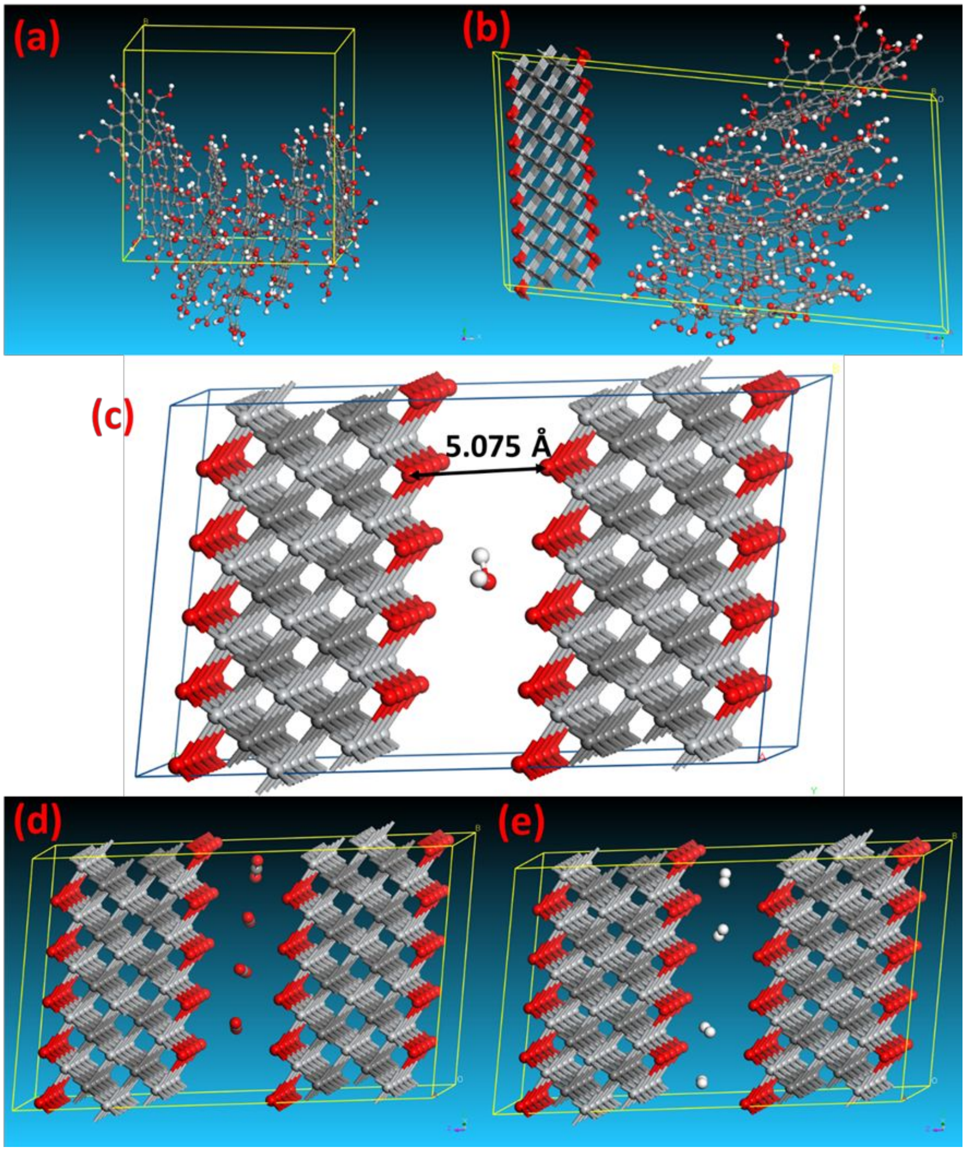

Figure S2. Prepared simulation interfaces: (a) GO–GO interface, (b) GO-$Ti_3C_2O_2$ composite interface, (c) $Ti_3C_2O_2$ membrane with one H_2O molecule in its interlayer space, (d) $Ti_3C_2O_2$ membrane with four CO_2 molecules in its interlayer space, (e) $Ti_3C_2O_2$ membrane with four H_2 molecules in its interlayer space. Color legend: Ti (light gray), C (dark gray), O (red), H (white), N (dark blue), F (light blue).

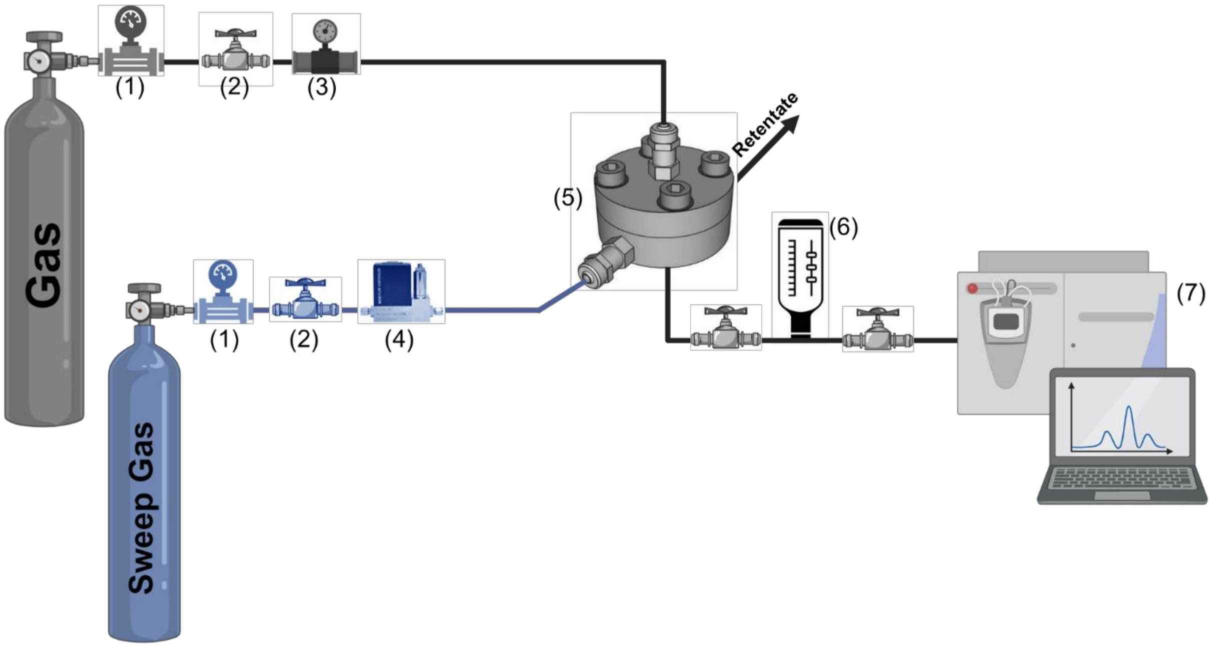

Figure S3. Schematic illustration of the gas permeation set-up. (1) pressure regulator, (2) valve, (3) digital pressure-gauge, (4) mass flow controller, (5) membrane holder, (6) flow meter, and (7) gas chromatograph.

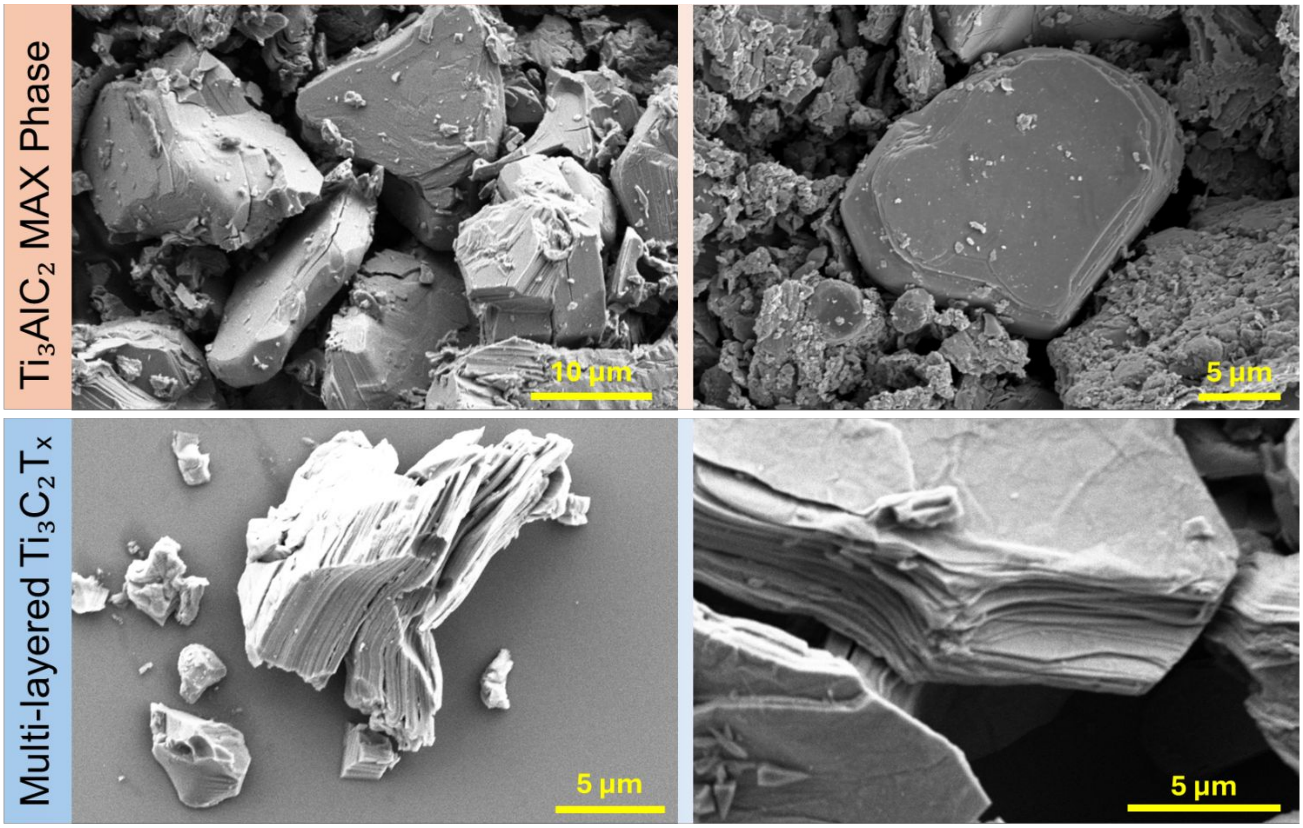

Figure S4. SEM images of bulk Ti_3AlC_2 MAX phase and multi-layered $Ti_3C_2T_x$ MXenes.

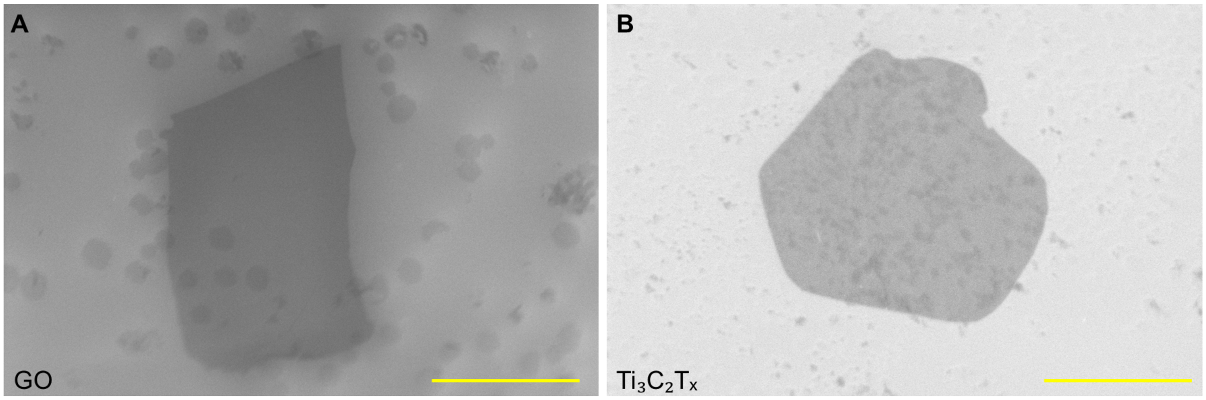

Figure S5. SEM image of single-layer (A) GO and (B) $Ti_3C_2T_x$ nanosheets. Scale bar: 4 μm.

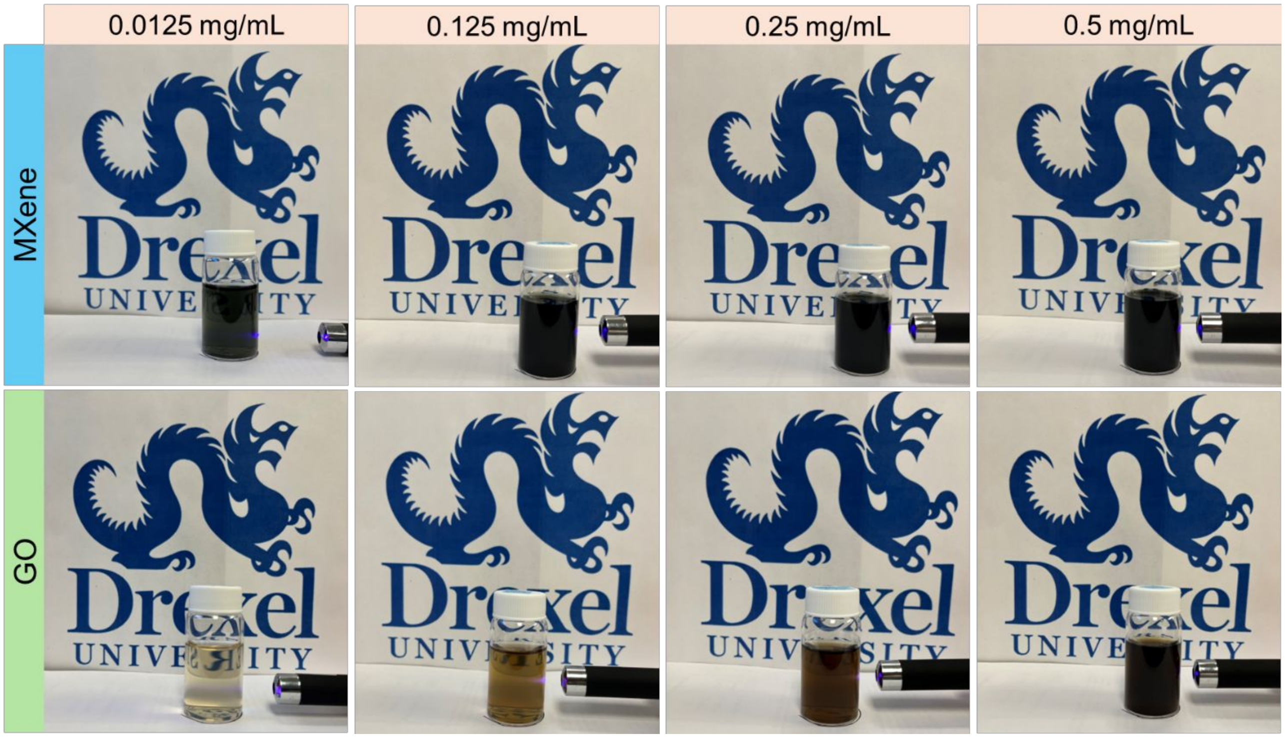

Figure S6. Tyndall scattering effect in suspensions with different concentrations.

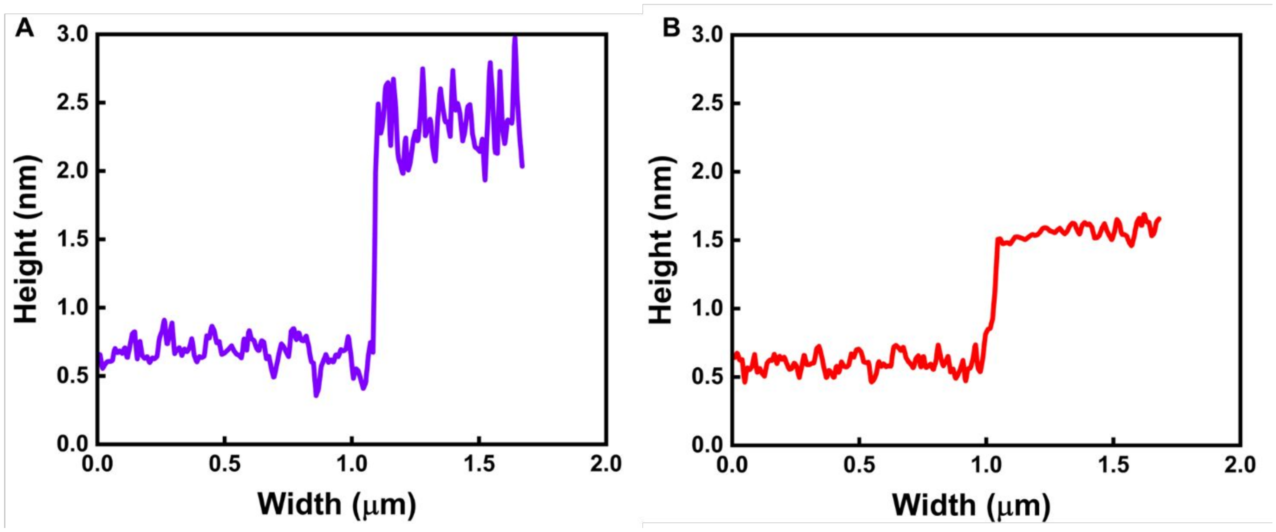

Figure S7. Height profiles of (A) $Ti_3C_2T_x$ and (B) GO nanosheets, extracted along the purple and red lines in the corresponding AFM topography images.

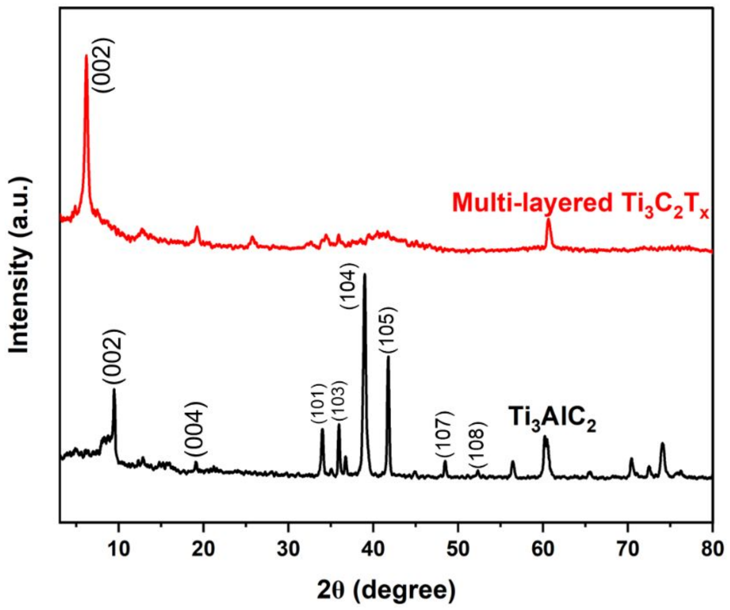

Figure S8. XRD patterns of the Ti_3AlC_2 MAX phase and multilayered $Ti_3C_2T_x$ MXene.

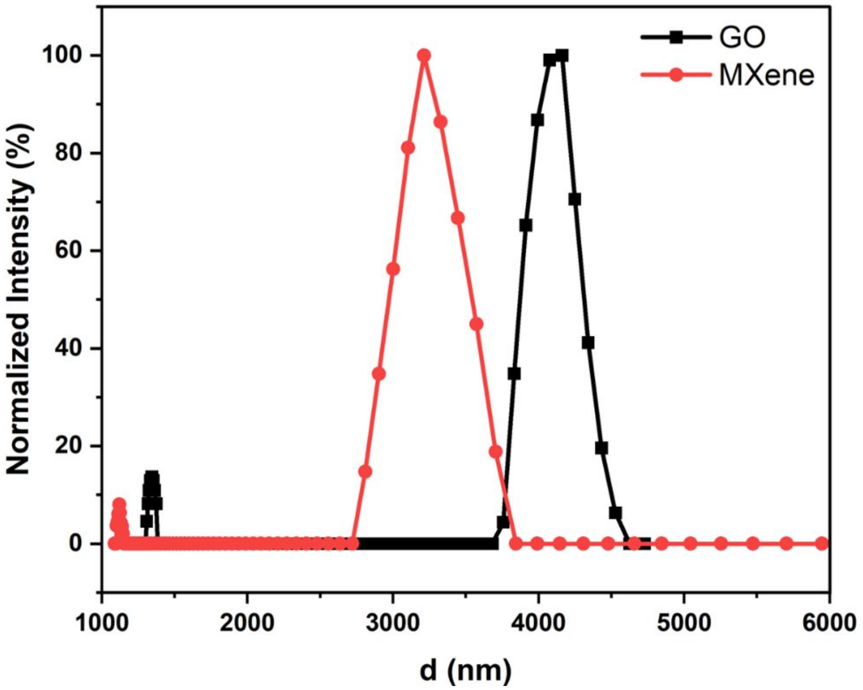

Figure S9. Particle size distributions of $Ti_3C_2T_x$ and GO nanosheets in aqueous suspensions, measured by DLS.

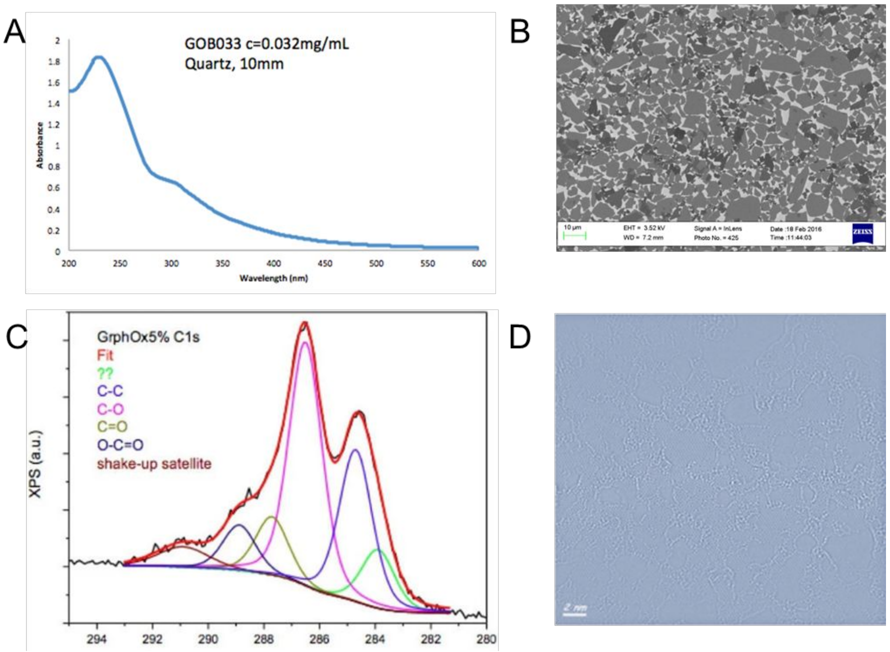

Figure S10. Supplier-provided characterization for graphene oxide (GO). (A) UV–Vis spectrum of an aqueous GO dispersion (quartz cuvette, 10 mm path length), showing the π–π* band near ~230 nm and an n–π* shoulder around ~300 nm. (B) SEM micrograph (scale bar: 10 µm). (C) High-resolution XPS C 1s spectrum deconvoluted into C–C/C=C, C–O, C=O, and O–C=O components with a π–π* shake-up satellite. (D) TEM micrograph (scale bar: 2 nm).

Supplementary Note 1

Figure S10A shows the UV-Vis analysis of GO. Aqueous dispersions of graphene oxide typically exhibit a strong absorption maximum near ~230 nm, attributed to the π–π* transition of aromatic C=C domains, together with a broad shoulder around ~300 nm arising from n–π* transitions of carbonyl and other oxygen containing functional groups. This spectral signature is characteristic of oxidized graphene sheets comprising sp^2 islands embedded in an oxygen-functionalized matrix.[1]

The supplier-provided SEM micrograph (Figure S10B) shows a polydisperse population of plate-like GO flakes with clean, polygonal edges and minimal extraneous debris. The darker

contrast regions correspond to thicker or overlapping areas, whereas brighter regions indicate thinner flakes and exposed substrate. While SEM does not quantify layer number, the observed morphology is consistent with monolayer-rich, well-exfoliated GO, as illustrated by the SEM image of a single flake in Figure S5A. Quantitative thickness is confirmed by AFM (Figure 2F and Figure S7B).

The high-resolution C 1s XPS spectrum of GO (Figure S10C) was deconvoluted into components assigned to sp^2 C–C, C–O (hydroxyl/epoxy), C=O (carbonyl), and O–C=O (carboxyl/ester). A weak π–π* shake-up feature is occasionally observed at higher binding energy, consistent with the presence of residual conjugated domains.[2]

The TEM micrograph (Figure S10D) shows electron-transparent GO nanosheets with locally ordered contrast features consistent with the hexagonal graphene lattice. Corrugation and wrinkling across the basal plane, together with occasional overlaps at flake junctions, are evident, and these features are typical of well-exfoliated, monolayer-rich GO.

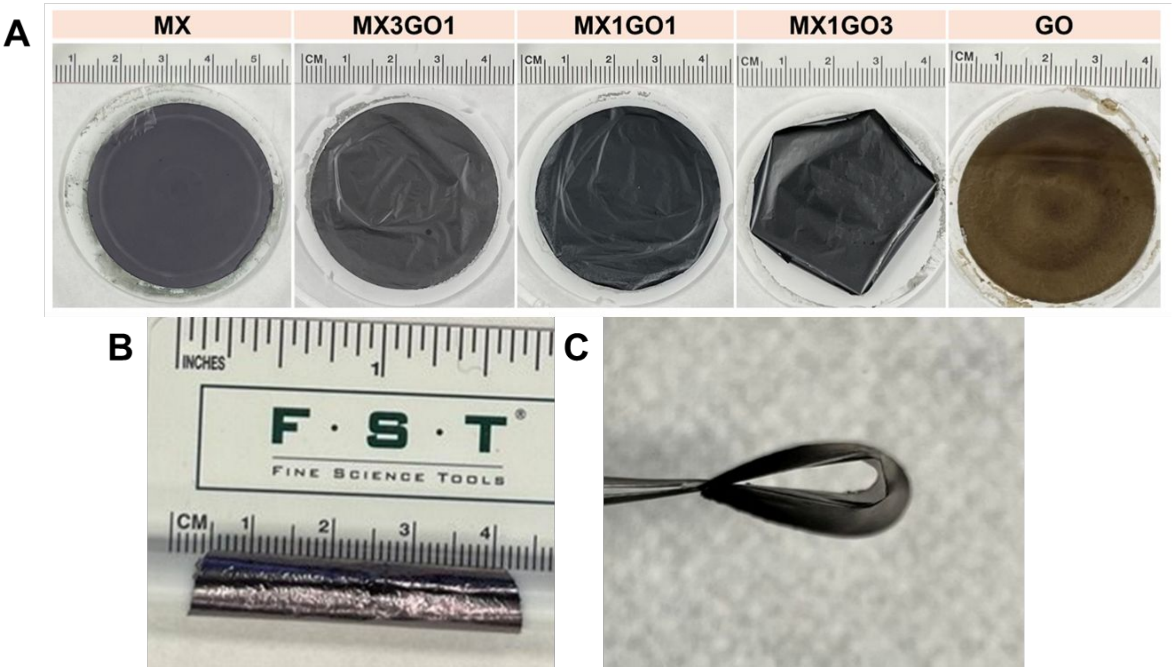

Figure S11. (A) Optimal images of the prepared membranes. MX1GO1 membrane was (B) wrapped around a rod and (C) flexed, demonstrating its excellent mechanical robustness.

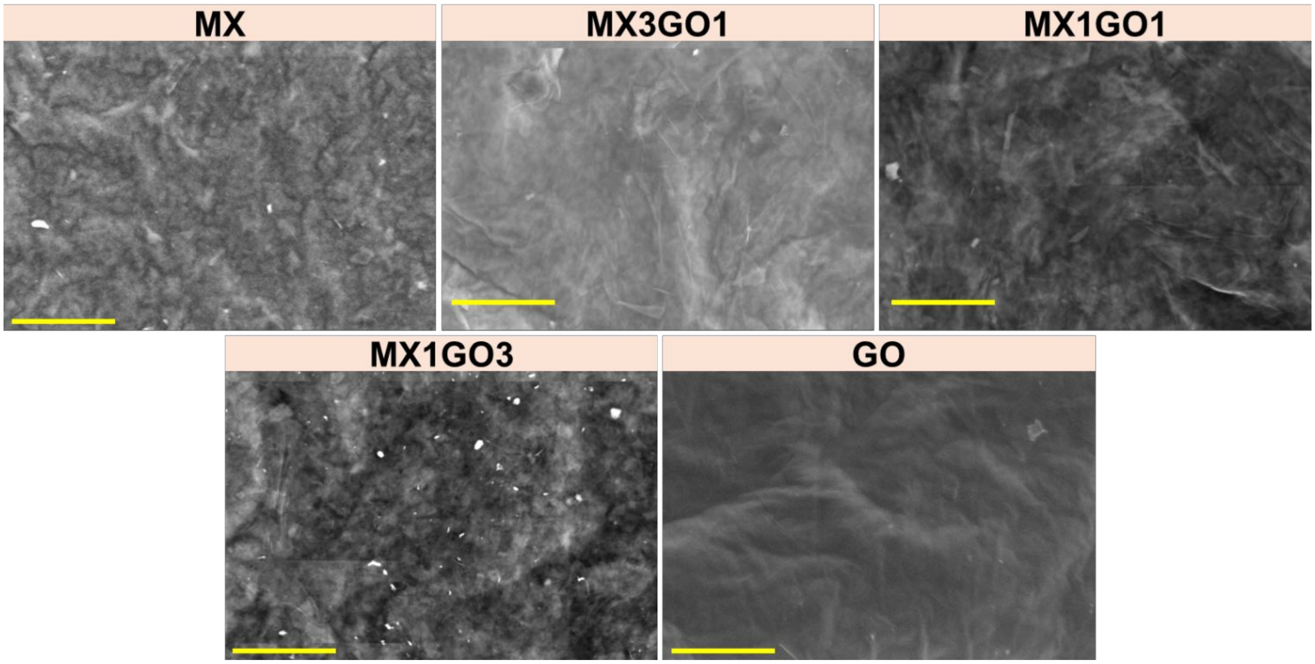

Figure S12. Top-view SEM images of pristine MXene, GO, and MXene/GO membranes. Scale bar: 3 μm.

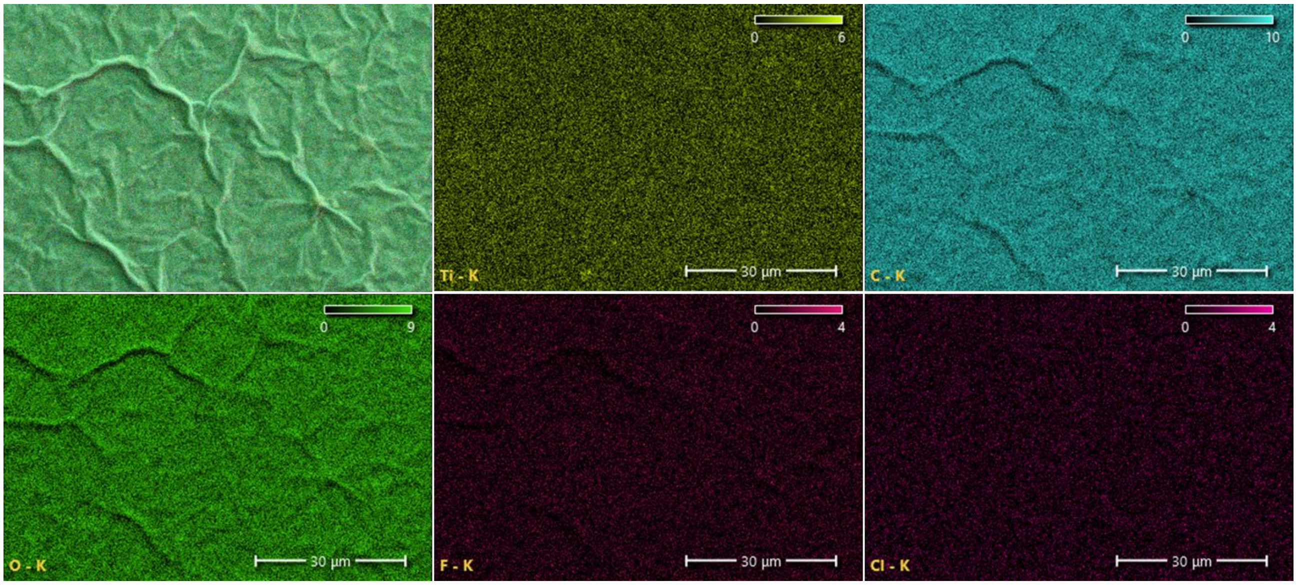

Figure S13. Elemental distribution mappings of the MX1GO1 membrane. Scale bar: 30 µm.

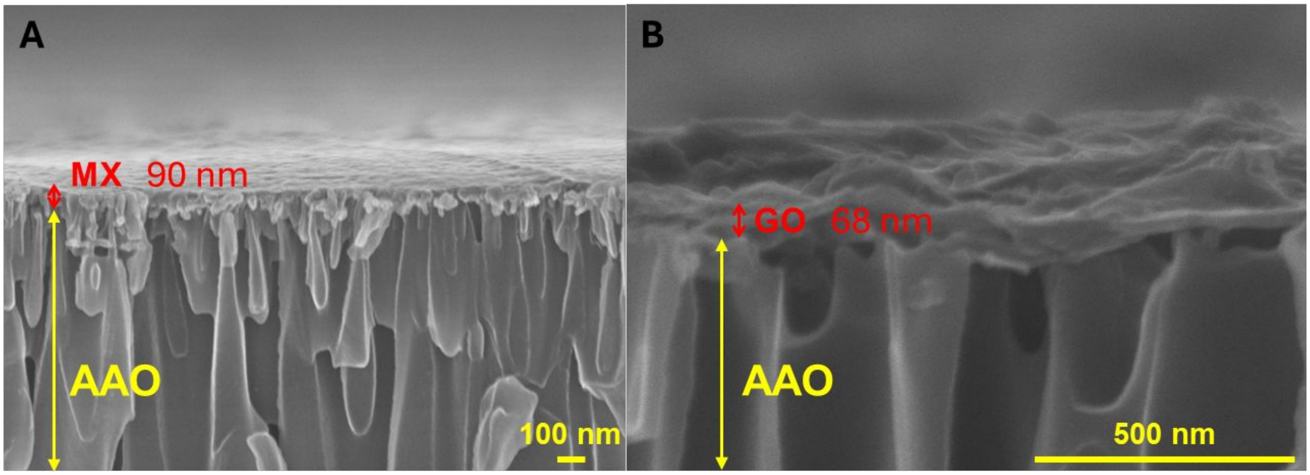

Figure S14. Cross-sectional SEM images of pristine (A) MXene and (B) GO membranes.

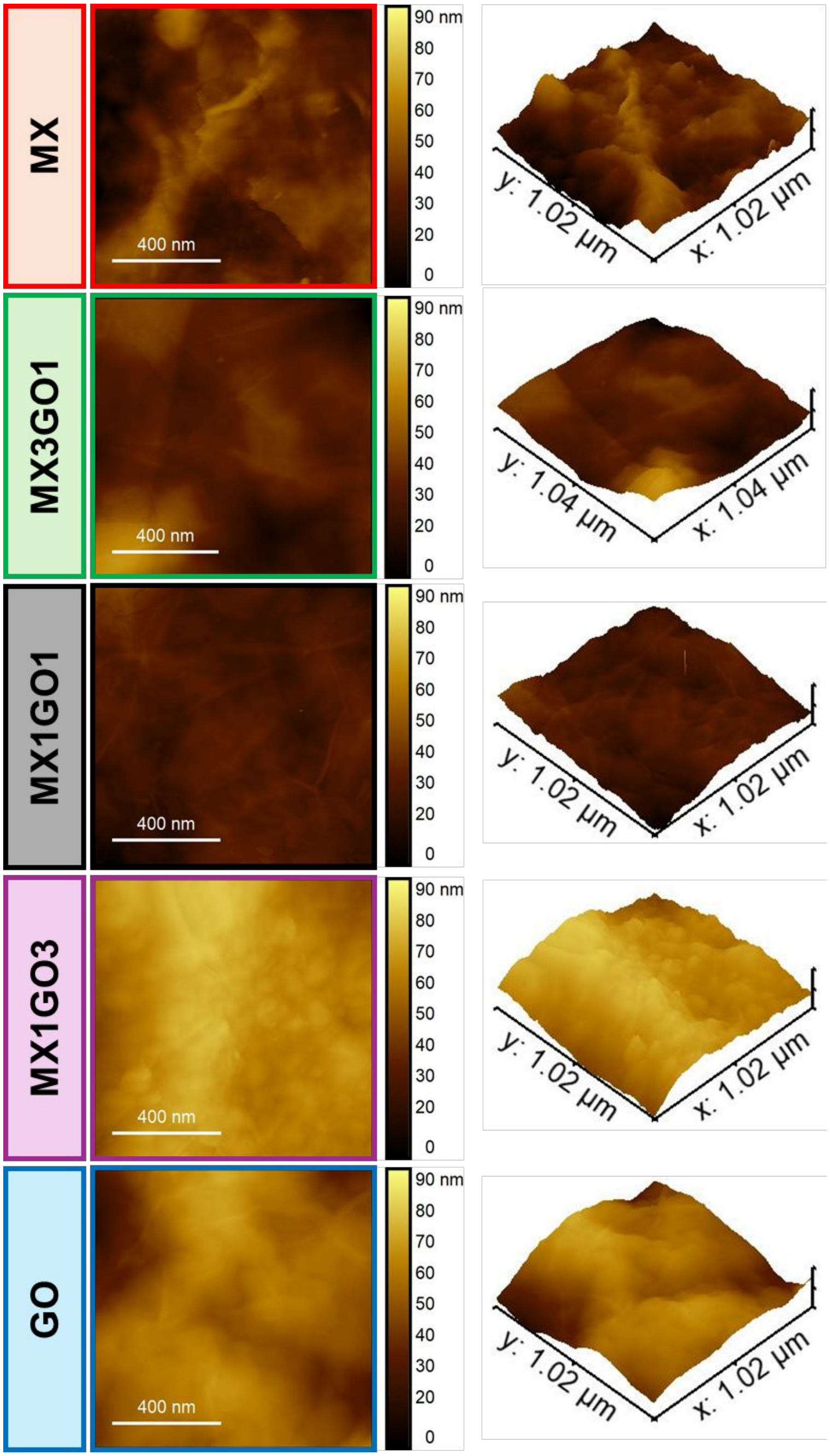

Figure S15. AFM topography of the membrane surfaces. Scale bar: 200 nm.

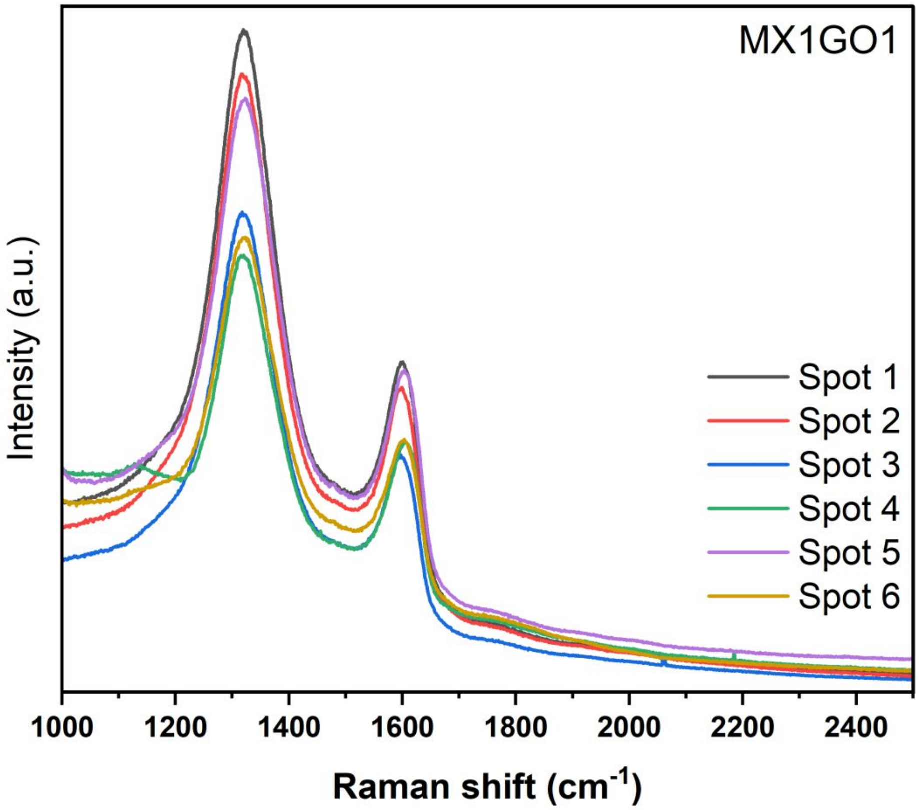

Figure S16. Raman spectra of the MX1GO1 membrane collected from six different locations on the same sample (D and G bands shown).

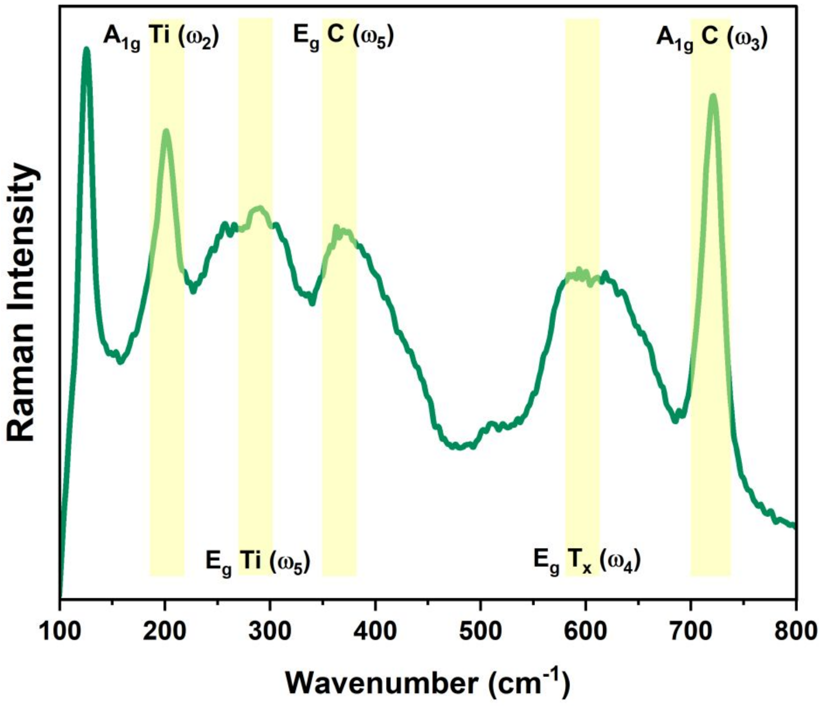

Figure S17. Raman spectrum of pristine $Ti_3C_2T_x$.

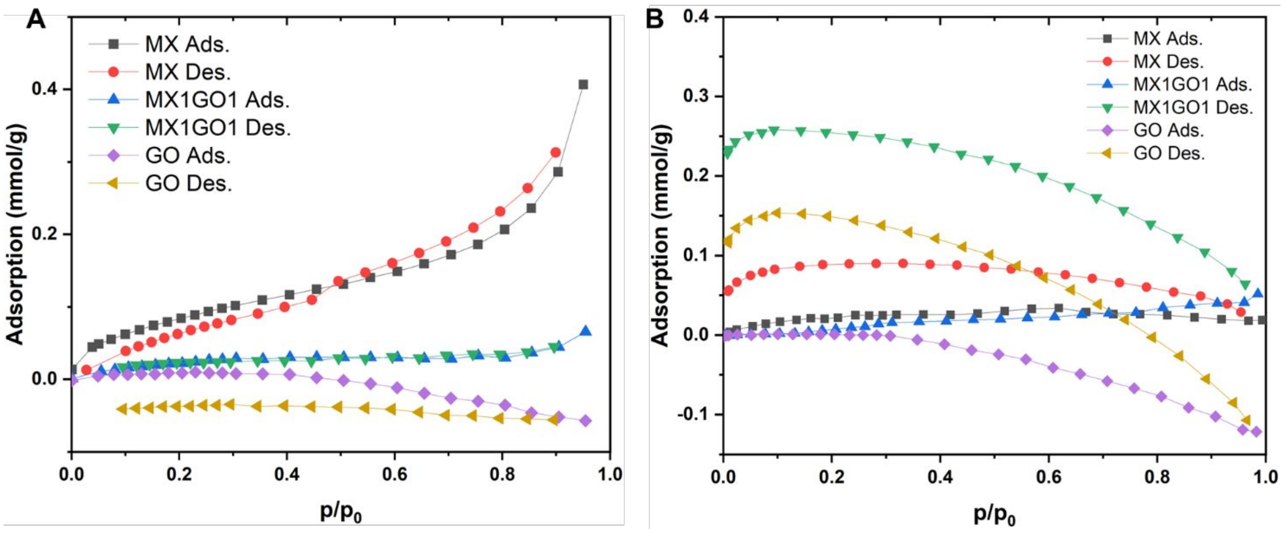

Figure S18. (A) N_2 and (B) CO_2 adsorption-desorption isotherms of pristine MXene, pristine GO, and MX1GO1 membranes at 77 K.

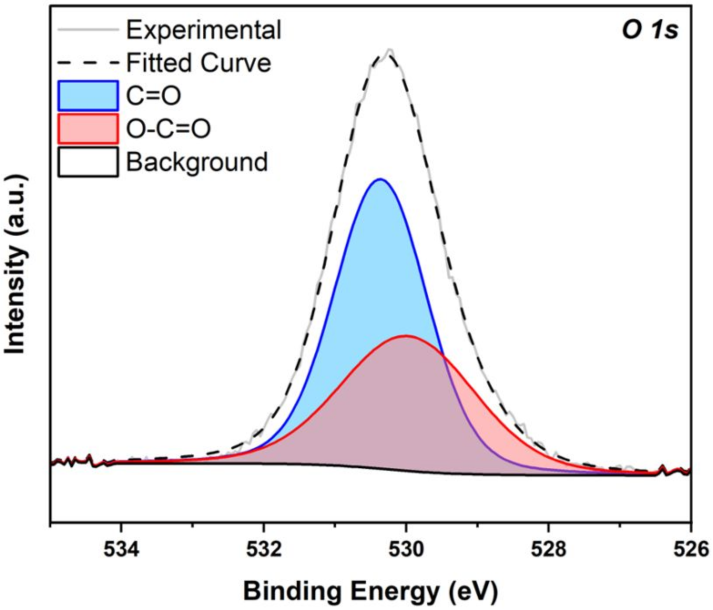

Figure S19. High-resolution O 1s XPS spectrum of the pristine GO membrane.

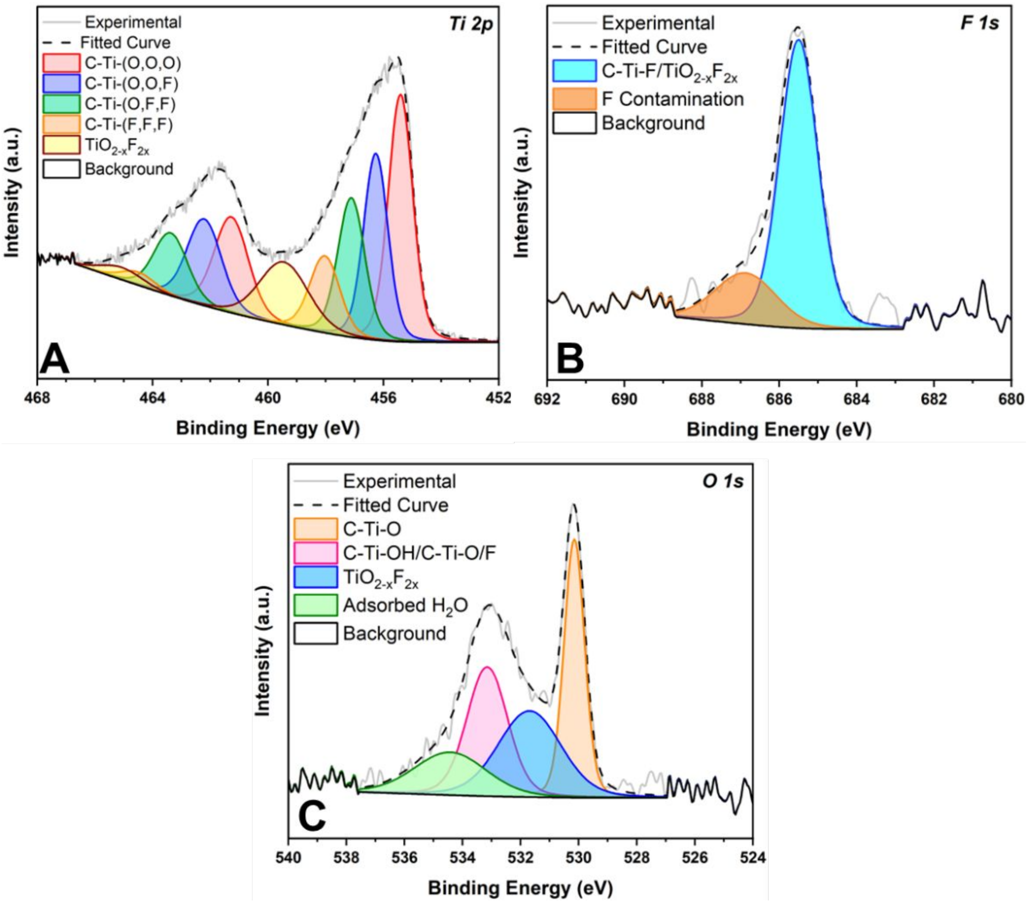

Figure S20. High-resolution XPS spectra of the pristine MXene membrane: (A) Ti 2p, (B) F 1s, and (C) O 1s regions.

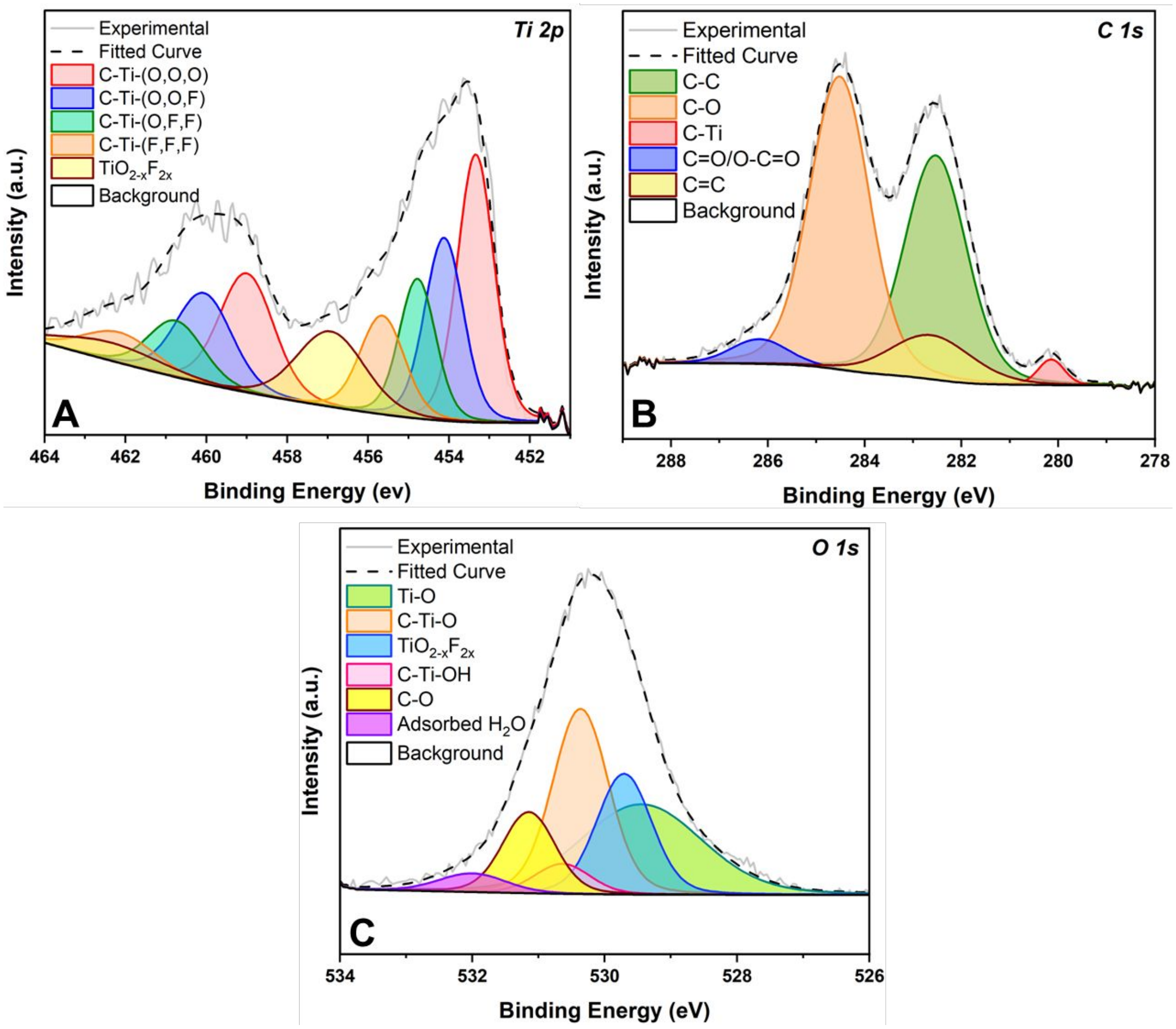

Figure S21. High-resolution XPS spectra of the MX1GO3 membrane: (A) Ti 2p, (B) C 1s, and (C) O 1s regions.

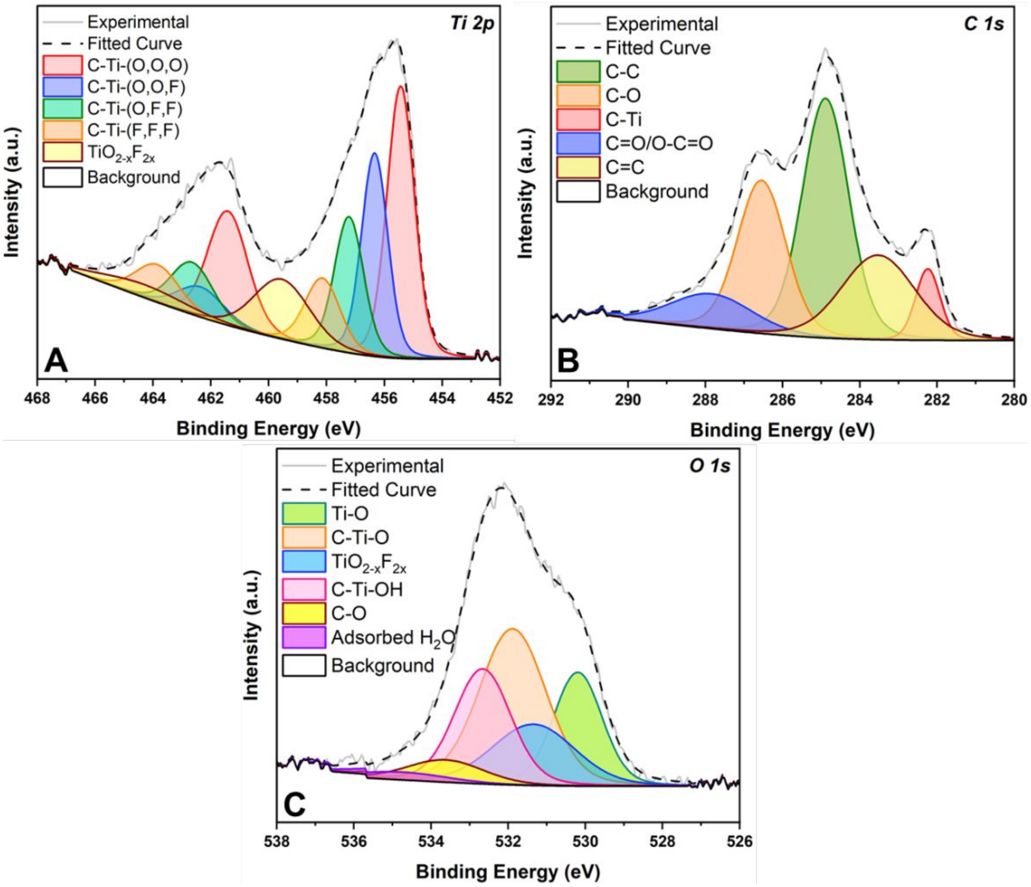

Figure S22. High-resolution XPS spectra of the MX1GO1 membrane: (A) Ti 2p, (B) C 1s, and (C) O 1s regions.

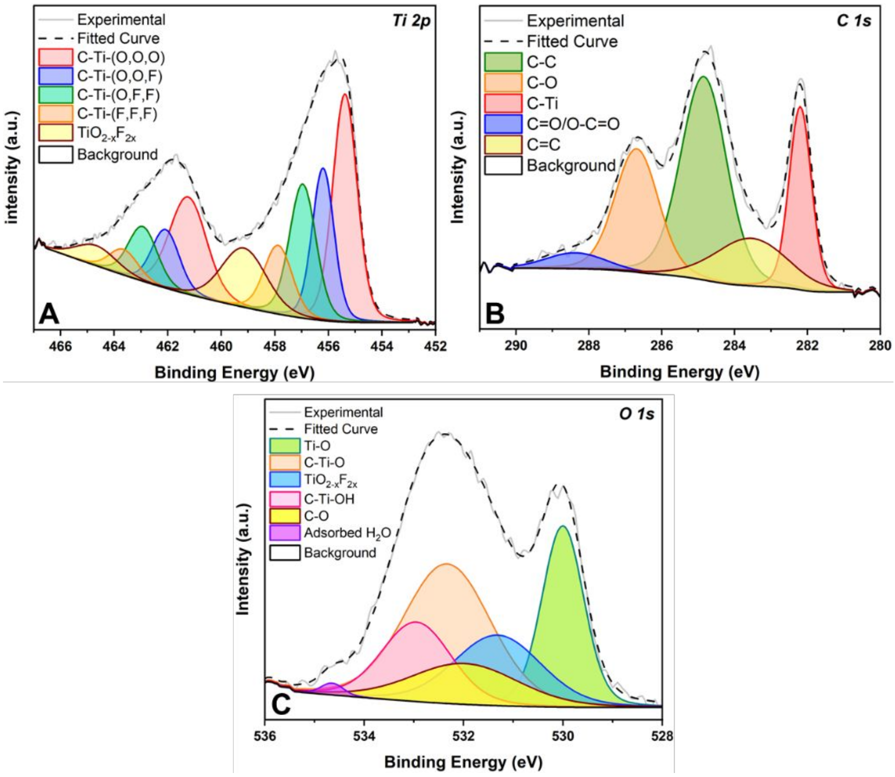

Figure S23. High-resolution XPS spectra of the MX3GO1 membrane: (A) Ti 2p, (B) C 1s, and (C) O 1s regions.

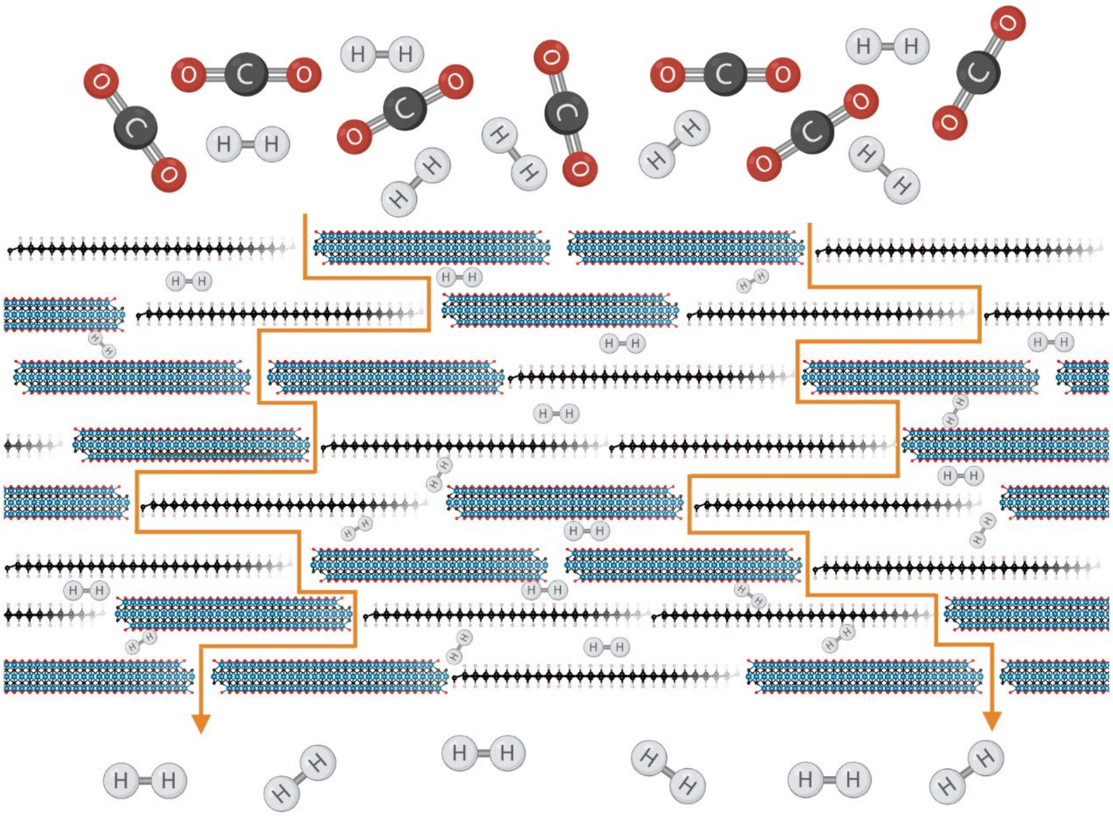

Figure S24. Schematic of molecular transport in MXene/GO membranes, showing H_2 and CO_2 molecules traversing slit-like pores and interlayer galleries between adjacent MXene and GO nanosheets.

Table S1. Supplier quality-assurance data for graphene oxide (GO) used in this study.

Metric	Value
Color	Dark brown
Solvent	Water
Monolayer Content	>95%
Elemental composition (wt%)	C 49-56<br>O 41-50<br>H 1-2<br>N 0-1<br>S 2-3
Derived C/O ratio (atomic)	1.3-1.8

Table S2. Surface roughness parameters of the membranes.

Membrane	Roughness parameters	
	R_q (nm)	R_a (nm)
MX	10.41	8.41
MX3GO1	10.54	7.28
MX1GO1	4.92	3.90
MX1GO3	7.32	5.87
GO	10.51	8.09

Table S3. *d*-spacing of pristine and composite membranes (from XRD).

Membrane	*d*-spacing (nm)
MX	1.54
MX3GO1	1.34
MX1GO1	1.16
MX1GO3	1.24
GO	0.80

Table S4. BET analysis of pristine and composite membranes.

Membrane	Surface area (m^2/g)	V_m (cm^3 (STP)/g)	D_p (nm)
MX	7.56	1.73	7.45
GO	0.81	0.18	0.96
MX1GO1	2.30	0.52	3.94

Table S5. Gas transport properties of the membranes at 1 bar and room temperature.

Membrane	Permeance (GPU)					Selectivity			
	H_2	CO_2	CH_4	C_2H_4	C_2H_6	H_2/CO_2	H_2/CH_4	H_2/C_2H_4	H_2/C_2H_6
MX	7850.4	68.2	302.9	36.1	46.0	115.1	25.9	217.4	170.6
MX3GO1	3840.6	18.5	123.1	11.1	14.4	207.6	31.2	346.0	266.7
MX1GO1	2681.6	5.0	48.2	3.1	4.1	536.3	55.6	865.1	654.1
MX1GO3	1492.6	4.1	29.7	2.8	3.5	364.0	50.2	533.0	426.4
GO	821.2	2.7	20.3	1.7	2.1	304.1	40.4	483.1	391.0

Table S6. Comparison of H_2/CO_2 separation performances between this work and previously reported membranes.

Category	Number	Membrane Material	Temperature (°C)	H_2/CO_2 Ratio	H_2 Permeance (GPU)	Separation factor (H_2/CO_2)	Ref.
Zeolites	1	NaA	25	50/50	406	7	3
	2	LTA	30	50/50	7250	15	4
	3	ZSM-5-Silicate	450	50/50	371	25	5
	4	RUB-15	250	50/50	848	154	6
COFs	5	TpPaMe	RT	50/50	727	13	7
	6	TpTG@TpPa/COF-LZU1	RT	50/50	2163	26	8
	7	CTF-BTD	25	50/50	655	43.1	9
	8	COF-LZU1	25	50/50	3600	32	10
	9	TpEBr@TpPa-SO_3Na	25	50/50	2566	23	11
	10	COF-LZU1-ACOF-1	25	50/50	710.5	27	12
MOFs	11	ZIF-L	25	50/50	4033	321	13
	12	bMOF-201	RT	50/50	1877	22	14
	13	ZIF-95	RT	50/50	2291	32	15
	14	ZIF-8	25	50/50	4495	3	16
	15	ZIF-95	RT	50/50	493	42	17
	16	NH_2-UiO-66	20	50/50	1039	29	18
	17	Anth	25	50/50	71.9	32	19
	18	ZIF-8	25	20/80	426	70	20
	19	$Zn_2(bim)_4$	30	50/50	406	106	21
	20	$Zn_2(bim)_3$	20	50/50	2320	166	22
	21	UiO-67	RT	50/50	1810	9	23
	22	MAMS-1	RT	50/50	715	245	24
	23	$Co_3(HCOO)_6$	RT	N/A	6206	10	25
	24	$Zn_2(bim)_4$	RT	50/50	2547	261	26
	25	NH_2-MIL-53(Al)	15	50/50	5757	31	27
	26	NH_2-MIL-53(Ti)	30	50/50	129	23	28
	27	ZIF-8	25	50/50	406	4	29
	28	ZIF-67-in-TpPa-1	25	50/50	3553	33	30
	29	ZIF-8@GO	250	50/50	377	15	31
	30	$Zn_2(Bim)_4$ SSCMs	25	50/50	1313	1084	32
Polymers	32	TB-OR	35	N/A	0.5	5	33
	33	M-PBI	180	50/50	49	34	34
	34	PBI/sPPSU	90	50/50	17	10	35
	35	PBDI	150	50/50	241	23	36
	36	PIM-EA(H_2)-TB/PBI	250	50/50	58	24	37
	37	MPD-TMC	140	50/50	350	50	38
	38	CP glass	25	50/50	715	91	39
	39	BILP-101_x	150	50/50	24	40	40

Table S7. Qualitative comparison of $Ti_3C_2T_x$/GO composite membrane reports and this work.

System	Regime	Structural Control	Transport Behavior	Key Distinction vs This Work	Ref.
$Ti_3C_2T_x$/GO	Liquid (dyes)	Ratio-tuned galleries; chemistry affects spacing	Low-friction capillary channels; molecular sieving; electrostatic interaction	Liquid-phase only; no gas-phase H_2/CO_2 or mixed-gas validation; mechanism framed for aqueous transport	41
$Ti_3C_2T_x$/GO	Liquid (dyes)	Defect sealing (inter-edge pathways)	molecular sieving; electrostatic interaction; liquid polarity effects	Defect sealing demonstrated in liquids; does not establish gas-phase H_2/CO_2 transport	42
$Ti_3C_2T_x$/GO+ urea	Liquid (dyes/ions)	Crosslinking for Å-level spacing control and stability	Molecular/ion sieving in water	Crosslinking-based aqueous sieving; no gas-phase H_2/CO_2 or mixed-gas validation	43
$Ti_3C_2T_x$/GO+ PEI/PDA	Liquid (metal ions)	Charge tuning; surface modification	Donnan exclusion; Steric effect	Ion/charge-dominated aqueous separation; different transport physics than gas-phase H_2/CO_2	44
$Ti_3C_2T_x$/GO	Liquid (OSN & dyes)	Composition-tuned spacing	Molecular Sieving; adsorption; Low-friction capillary channels	OSN/liquid separation focus; no gas-phase H_2/CO_2 or mixed-gas validation	45
$Ti_3C_2T_x$/rGO	Gas (H_2/CO_2; pure)	Orientation control (vertical vs horizontal)	Short Path Transport; temperature effects	Architecture relies on orientation engineering rather than GO-driven defect sealing	46
$Ti_3C_2T_x$/GO	Gas (H_2/CO_2; pure and mixed)	GO-driven defect sealing; ratio-tuned packing/gallery regulation; interface effects	Molecular sieving-dominated; tortuosity; quadrupolar interactions	Gas-phase $Ti_3C_2T_x$/GO with mixed-gas validation; systematic ratio-structure-transport framework	This work

Table S8. Confined diffusion coefficients and H_2/CO_2 diffusion selectivity at the material interface.

Membrane	H_2 diffusion coefficient (10^{-8} m^2/s)	CO_2 diffusion coefficient (10^{-8} m^2/s)	H_2/CO_2 selectivity
$Ti_3C_2O_2$	11.39	0.18	61.29
GO	2.72	0.08	31.49
GO-$Ti_3C_2O_2$ composite	18.66	0.08	209.60

References

(1) Lai, Q.; Zhu, S.; Luo, X.; Zou, M.; Huang, S. Ultraviolet-visible spectroscopy of graphene oxides. *AIP advances* **2012**, *2* (3).
(2) Al-Gaashani, R.; Najjar, A.; Zakaria, Y.; Mansour, S.; Atieh, M. XPS and structural studies of high quality graphene oxide and reduced graphene oxide prepared by different chemical oxidation methods. *Ceramics International* **2019**, *45* (11), 14439-14448.
(3) Wei, X.-L.; Pan, W.-Y.; Pan, M.; Peng, L.-L.; Niu, C.-Q.; Chao, Z.-S. Effects of bubbles on the structure and performance of zeolite membranes. *Journal of the European Ceramic Society* **2020**, *40* (4), 1709-1716.
(4) Sen, M.; Dana, K.; Das, N. Development of LTA zeolite membrane from clay by sonication assisted method at room temperature for H2-CO2 and CO2-CH4 separation. *Ultrasonics sonochemistry* **2018**, *48*, 299-310.
(5) Wang, H.; Lin, Y. Synthesis and modification of ZSM-5/silicalite bilayer membrane with improved hydrogen separation performance. *J. Membr. Sci.* **2012**, *396*, 128-137.
(6) Dakhchoune, M.; Villalobos, L. F.; Semino, R.; Liu, L.; Rezaei, M.; Schouwink, P.; Avalos, C. E.; Baade, P.; Wood, V.; Han, Y. Gas-sieving zeolitic membranes fabricated by condensation of precursor nanosheets. *Nat. Mater.* **2021**, *20* (3), 362-369.
(7) Zheng, W.; Hou, J.; Liu, C.; Liu, P.; Li, L.; Chen, L.; Tang, Z. Melamine-Doped Covalent Organic Framework Membranes for Enhanced Hydrogen Purification. *Chemistry–An Asian Journal* **2021**, *16* (22), 3624-3629.
(8) Ying, Y.; Peh, S. B.; Yang, H.; Yang, Z.; Zhao, D. Ultrathin covalent organic framework membranes via a multi-interfacial engineering strategy for gas separation. *Adv. Mater.* **2022**, *34* (25), 2104946.
(9) Zhao, Y.; Liu, P.; Ying, Y.; Wei, K.; Zhao, D.; Liu, D. Heating-driven assembly of covalent organic framework nanosheets for gas separation. *J. Membr. Sci.* **2021**, *632*, 119326.
(10) Fan, H.; Peng, M.; Strauss, I.; Mundstock, A.; Meng, H.; Caro, J. r. High-flux vertically aligned 2D covalent organic framework membrane with enhanced hydrogen separation. *J. Am. Chem. Soc.* **2020**, *142* (15), 6872-6877.
(11) Ying, Y.; Tong, M.; Ning, S.; Ravi, S. K.; Peh, S. B.; Tan, S. C.; Pennycook, S. J.; Zhao, D. Ultrathin two-dimensional membranes assembled by ionic covalent organic nanosheets with reduced apertures for gas separation. *J. Am. Chem. Soc.* **2020**, *142* (9), 4472-4480.
(12) Fan, H.; Mundstock, A.; Feldhoff, A.; Knebel, A.; Gu, J.; Meng, H.; Caro, J. r. Covalent organic framework–covalent organic framework bilayer membranes for highly selective gas separation. *J. Am. Chem. Soc.* **2018**, *140* (32), 10094-10098.
(13) Yang, K.; Hu, S.; Ban, Y.; Zhou, Y.; Cao, N.; Zhao, M.; Xiao, Y.; Li, W.; Yang, W. ZIF-L membrane with a membrane-interlocked-support composite architecture for H2/CO2 separation. *Science Bulletin* **2021**, *66* (18), 1869-1876.
(14) Schulte, Z. M.; Kwon, Y. H.; Han, Y.; Liu, C.; Li, L.; Yang, Y.; Jarvi, A. G.; Saxena, S.; Veser, G.; Johnson, J. K. H 2/CO 2 separations in multicomponent metal-adeninate MOFs with multiple chemically distinct pore environments. *Chemical Science* **2020**, *11* (47), 12807-12815.
(15) Ma, X.; Wan, Z.; Li, Y.; He, X.; Caro, J.; Huang, A. Anisotropic gas separation in oriented ZIF-95 membranes prepared by vapor-assisted in-plane epitaxial growth. *Ed. Angew. Chem., Int. Ed.* **2020**, *59* (47), 20858-20862.

(16) Hao, J.; Babu, D. J.; Liu, Q.; Chi, H.-Y.; Lu, C.; Liu, Y.; Agrawal, K. V. Synthesis of high-performance polycrystalline metal–organic framework membranes at room temperature in a few minutes. *J. Mater. Chem. A* **2020**, *8* (16), 7633-7640.
(17) Ma, X.; Li, Y.; Huang, A. Synthesis of nano-sheets seeds for secondary growth of highly hydrogen permselective ZIF-95 membranes. *J. Membr. Sci.* **2020**, *597*, 117629.
(18) Sun, Y.; Song, C.; Guo, X.; Liu, Y. Concurrent manipulation of out-of-plane and regional in-plane orientations of NH2-UiO-66 membranes with significantly reduced anisotropic grain boundary and superior H2/CO2 separation performance. *ACS Appl. Mater. Interfaces.* **2019**, *12* (4), 4494-4500.
(19) Ghalei, B.; Wakimoto, K.; Wu, C. Y.; Isfahani, A. P.; Yamamoto, T.; Sakurai, K.; Higuchi, M.; Chang, B. K.; Kitagawa, S.; Sivaniah, E. Rational tuning of zirconium metal–organic framework membranes for hydrogen purification. *Ed. Angew. Chem., Int. Ed.* **2019**, *58* (52), 19034-19040.
(20) Hou, J.; Hong, X.; Zhou, S.; Wei, Y.; Wang, H. Solvent-free route for metal–organic framework membranes growth aiming for efficient gas separation. *AIChE Journal* **2019**, *65* (2), 712-722.
(21) Li, Y.; Liu, H.; Wang, H.; Qiu, J.; Zhang, X. GO-guided direct growth of highly oriented metal–organic framework nanosheet membranes for H 2/CO 2 separation. *Chemical science* **2018**, *9* (17), 4132-4141.
(22) Peng, Y.; Li, Y.; Ban, Y.; Yang, W. Two-dimensional metal–organic framework nanosheets for membrane-based gas separation. *Angew. Chem.* **2017**, *129* (33), 9889-9893.
(23) Knebel, A.; Sundermann, L.; Mohmeyer, A.; Strauß, I.; Friebe, S.; Behrens, P.; Caro, J. r. Azobenzene guest molecules as light-switchable CO2 valves in an ultrathin UiO-67 membrane. *Chem. Mat.* **2017**, *29* (7), 3111-3117.
(24) Wang, X.; Chi, C.; Zhang, K.; Qian, Y.; Gupta, K. M.; Kang, Z.; Jiang, J.; Zhao, D. Reversed thermo-switchable molecular sieving membranes composed of two-dimensional metal-organic nanosheets for gas separation. *Nat. Commun.* **2017**, *8* (1), 14460.
(25) Sun, Y.; Yang, F.; Wei, Q.; Wang, N.; Qin, X.; Zhang, S.; Wang, B.; Nie, Z.; Ji, S.; Yan, H. Oriented Nano-Microstructure-Assisted Controllable Fabrication of Metal-Organic Framework Membranes on Nickel Foam. *Advanced Materials (Deerfield Beach, Fla.)* **2016**, *28* (12), 2374-2381.
(26) Peng, Y.; Li, Y.; Ban, Y.; Jin, H.; Jiao, W.; Liu, X.; Yang, W. Metal-organic framework nanosheets as building blocks for molecular sieving membranes. *Science* **2014**, *346* (6215), 1356-1359.
(27) Zhang, F.; Zou, X.; Gao, X.; Fan, S.; Sun, F.; Ren, H.; Zhu, G. Hydrogen selective NH2-MIL-53 (Al) MOF membranes with high permeability. *Adv. Funct. Mater.* **2012**, *22* (17), 3583-3590.
(28) Sun, Y.; Liu, Y.; Caro, J.; Guo, X.; Song, C.; Liu, Y. In-plane epitaxial growth of highly c-oriented NH2-MIL-125 (Ti) membranes with superior H2/CO2 selectivity. *Ed. Angew. Chem., Int. Ed.* **2018**, *57* (49), 16088-16093.
(29) Liu, Y.; Wang, N.; Pan, J. H.; Steinbach, F.; Caro, J. r. In situ synthesis of MOF membranes on ZnAl-CO3 LDH buffer layer-modified substrates. *J. Am. Chem. Soc.* **2014**, *136* (41), 14353-14356.
(30) Fan, H.; Peng, M.; Strauss, I.; Mundstock, A.; Meng, H.; Caro, J. MOF-in-COF molecular sieving membrane for selective hydrogen separation. *Nat. Commun.* **2021**, *12* (1), 38.

(31) Huang, A.; Liu, Q.; Wang, N.; Zhu, Y.; Caro, J. r. Bicontinuous zeolitic imidazolate framework ZIF-8@ GO membrane with enhanced hydrogen selectivity. *J. Am. Chem. Soc.* **2014**, *136* (42), 14686-14689.
(32) Shu, L.; Peng, Y.; Yao, R.; Song, H.; Zhu, C.; Yang, W. Flexible soft-solid metal–organic framework composite membranes for H2/CO2 separation. *Ed. Angew. Chem., Int. Ed.* **2022**, *61* (14), e202117577.
(33) Wu, L.; Chen, X.; Zhang, Z.; Xu, S.; Ma, C.; Li, N. Enhanced molecular selectivity and plasticization resistance in ring-opened Tröger's base polymer membranes. *J. Membr. Sci.* **2021**, *634*, 119399.
(34) Sánchez-Laínez, J.; Etxeberria-Benavides, M.; David, O.; Téllez, C.; Coronas, J. Green preparation of thin films of polybenzimidazole on flat and hollow fiber supports: application to hydrogen separation. *ChemSusChem* **2021**, *14* (3), 952-960.
(35) Naderi, A.; Chung, T.-S.; Weber, M.; Maletzko, C. High performance dual-layer hollow fiber membrane of sulfonated polyphenylsulfone/polybenzimidazole for hydrogen purification. *J. Membr. Sci.* **2019**, *591*, 117292.
(36) Shan, M.; Liu, X.; Wang, X.; Liu, Z.; Iziyi, H.; Ganapathy, S.; Gascon, J.; Kapteijn, F. Novel high performance poly (p-phenylene benzobisimidazole)(PBDI) membranes fabricated by interfacial polymerization for H 2 separation. *J. Mater. Chem. A* **2019**, *7* (15), 8929-8937.
(37) Sánchez-Laínez, J.; Zornoza, B.; Carta, M.; Malpass-Evans, R.; McKeown, N. B.; Téllez, C.; Coronas, J. Hydrogen separation at high temperature with dense and asymmetric membranes based on PIM-EA (H2)-TB/PBI blends. *Ind. Eng. Chem. Res.* **2018**, *57* (49), 16909-16916.
(38) Ali, Z.; Pacheco, F.; Litwiller, E.; Wang, Y.; Han, Y.; Pinnau, I. Ultra-selective defect-free interfacially polymerized molecular sieve thin-film composite membranes for H 2 purification. *J. Mater. Chem. A* **2018**, *6* (1), 30-35.
(39) Li, J.; Wang, J.; Li, Q.; Zhang, M.; Li, J.; Sun, C.; Yuan, S.; Feng, X.; Wang, B. Coordination Polymer Glasses with Lava and Healing Ability for High-Performance Gas Sieving. *Ed. Angew. Chem., Int. Ed.* **2021**, *60* (39), 21304-21309. DOI: https://doi.org/10.1002/anie.202102047.
(40) Shan, M.; Liu, X.; Wang, X.; Yarulina, I.; Seoane, B.; Kapteijn, F.; Gascon, J. Facile manufacture of porous organic framework membranes for precombustion CO₂ capture. *Sci. Adv.* **2018**, *4* (9), eaau1698. DOI: doi:10.1126/sciadv.aau1698.
(41) Liu, T.; Liu, X.; Graham, N.; Yu, W.; Sun, K. Two-dimensional MXene incorporated graphene oxide composite membrane with enhanced water purification performance. *J. Membr. Sci.* **2020**, *593*, 117431.
(42) Kang, K. M.; Kim, D. W.; Ren, C. E.; Cho, K. M.; Kim, S. J.; Choi, J. H.; Nam, Y. T.; Gogotsi, Y.; Jung, H.-T. Selective molecular separation on Ti3C2T x–graphene oxide membranes during pressure-driven filtration: comparison with graphene oxide and MXenes. *ACS Appl. Mater. Interfaces.* **2017**, *9* (51), 44687-44694.
(43) Yu, D.; Sun, L.; Zhang, Y.; Song, Y.; Jia, C.; Wang, Y.; Wang, Y.; Kipper, M. J.; Tang, J.; Huang, L. Two-dimensional graphene oxide/MXene lamellar membrane cross-linked by urea with adjustable interlayer spacing for efficient dye rejection and ion sieving. *Chem. Eng. J.* **2024**, *480*, 148009.
(44) Zhao, X.; Che, Y.; Mo, Y.; Huang, W.; Wang, C. Fabrication of PEI modified GO/MXene composite membrane and its application in removing metal cations from water. *J. Membr. Sci.* **2021**, *640*, 119847.

(45) Wei, S.; Xie, Y.; Xing, Y.; Wang, L.; Ye, H.; Xiong, X.; Wang, S.; Han, K. Two-dimensional graphene Oxide/MXene composite lamellar membranes for efficient solvent permeation and molecular separation. *J. Membr. Sci.* **2019**, *582*, 414-422.
(46) Dong, Z.; Fan, Y.; Meng, X.; Jin, Y.; Song, J.; Wang, X.; Yang, N.; Sunarso, J.; Liu, S. A vertically-stacked MXene/rGO composite membrane for highly efficient H 2/CO 2 separation. *Chem. Commun.* **2024**, *60* (39), 5177-5180.
